# Unraveling potential enzymes and their functional role in fine cocoa beans fermentation using temporal shotgun metagenomics

**DOI:** 10.3389/fmicb.2022.994524

**Published:** 2022-11-03

**Authors:** Carolina O. de C. Lima, Giovanni M. De Castro, Ricardo Solar, Aline B. M. Vaz, Francisco Lobo, Gilberto Pereira, Cristine Rodrigues, Luciana Vandenberghe, Luiz Roberto Martins Pinto, Andréa Miura da Costa, Maria Gabriela Bello Koblitz, Raquel Guimarães Benevides, Vasco Azevedo, Ana Paula Trovatti Uetanabaro, Carlos Ricardo Soccol, Aristóteles Góes-Neto

**Affiliations:** ^1^Department of Biological Sciences, State University of Feira de Santana (UEFS), Feira de Santana, Bahia, Brazil; ^2^Institute of Biological Sciences, Federal University of the Minas Gerais (UFMG), Belo Horizonte, Minas Gerais, Brazil; ^3^Bioprocess Engineering and Biotechnology Department, Federal University of the Paraná (UFPR), Curitiba, Paraná, Brazil; ^4^Department of Biological Sciences, State University of Santa Cruz (UESC), Ilhéus, Bahia, Brazil; ^5^Food and Nutrition Graduate Program (PPGAN), Federal University of the State of Rio de Janeiro (UNIRIO), Rio de Janeiro, Rio de Janeiro, Brazil

**Keywords:** *Theobroma cacao*, cocoa beans fermentation, ecological succession, microbiome, functional analysis

## Abstract

Cocoa beans fermentation is a spontaneous process, essential for the generation of quality starting material for fine chocolate production. The understanding of this process has been studied by the application of high-throughput sequencing technologies, which grants a better assessment of the different microbial taxa and their genes involved in this microbial succession. The present study used shotgun metagenomics to determine the enzyme-coding genes of the microbiota found in two different groups of cocoa beans varieties during the fermentation process. The statistical evaluation of the most abundant genes in each group and time studied allowed us to identify the potential metabolic pathways involved in the success of the different microorganisms. The results showed that, albeit the distinction between the initial (0 h) microbiota of each varietal group was clear, throughout fermentation (24–144 h) this difference disappeared, indicating the existence of selection pressures. Changes in the microbiota enzyme-coding genes over time pointed to the distinct ordering of fermentation at 24–48 h (T1), 72–96 h (T2), and 120–144 h (T3). At T1, the significantly more abundant enzyme-coding genes were related to threonine metabolism and those genes related to the glycolytic pathway, explained by the abundance of sugars in the medium. At T2, the genes linked to the metabolism of ceramides and hopanoids lipids were clearly dominant, which are associated with the resistance of microbial species to extreme temperatures and pH values. In T3, genes linked to trehalose metabolism, related to the response to heat stress, dominated. The results obtained in this study provided insights into the potential functionality of microbial community succession correlated to gene function, which could improve cocoa processing practices to ensure the production of more stable quality end products.

## Introduction

Chocolate is one of the most popular food products in the world. Its production depends on several stages, from the planting of cocoa trees to the molding of bars and chocolates (De Vuyst and Leroy, 2020). An important part of this process takes place on the farm and involves the spontaneous fermentation of the cocoa pulp, by a complex microbial succession, which allows the removal of the pulp and creates specific conditions within the cotyledons. These reactions promote chemical and enzymatic transformations that lead to the acquisition of sensory characteristics desired for chocolate (Lima et al., 2020). As it is a spontaneous process, cocoa beans fermentation may often not work as it should, generating low-quality products and creating a shortage of this commodity in the market (Verce et al., 2021).

Metagenomics has revolutionized our view of microbial ecology with many potential applications for biotechnology (Cuadros-Orellana et al., 2013). Usually, microbiome studies utilize short-read high-throughput sequencing (HTS) platforms (e.g.: Illumina) that generate very high yields but of short read lengths (150–300 base pairs; Fonseca et al., 2022), as the current study. Conversely, long-read HTS platforms (e.g.: Oxford Nanopore Technologies) can sequence long DNA segments (Kbp to Mbp; Tomé et al., 2022). Long-read sequencing may help not only in alignment-based taxonomic and functional assignment due to its increased information content but also in bridging within- and between-genome repetitive sequences (Wommack et al., 2008; Bertrand et al., 2018). Nonetheless, the aforementioned advantages are still impaired by a high error rate if compared to short-read sequencing and; therefore, the lower accuracy of long-read sequencing affects the success rate of current classification methods, as well as there are few algorithms specifically designed to exploit long-read data for metagenomics (Nicholls et al., 2019; Pearman et al., 2020). Furthermore, metagenomic studies using long-read sequencing has been increasing since last years but mainly limited to amplicon-based metagenomics targeted to investigate the structure (taxonomic composition and relative abundance) of microbial communities (Matsuo et al., 2021; Lu et al., 2022), and shotgun metagenomic studies are still quite scarce (Ciuffreda et al., 2021). Therefore, short-read HTS still remains the most advantageous platform to perform shotgun metagenomics.

In order to enable controlled fermentation, extensive research has been carried out to elucidate the processes involved in fermentation and understand which events are desirable and which are unwanted or unnecessary (Schwan and Wheals, 2004; De Vuyst and Leroy, 2020). The most recent efforts have been applying metagenomic technologies to unravel the microbiota involved in fermentation, its succession, and, more importantly, the role of each taxonomic group in the transformations that occur throughout the process (Illeghems et al., 2015; Lima et al., 2020; Verce et al., 2021). Among the main advances of this approach were (1) the confirmation of the wide diversity of fermentative communities, previously under- elucidated by culture-dependent methods, including heretofore unaccounted groups, such as the Enterobacteriaceae (Illeghems et al., 2015); (2) the further clarification of the role of microbial succession and the groups involved in raw material consumption and environmental transformations, including the discovery that the desired transformations may happen even in the absence of some of the microbial groups formerly considered indispensable (Lima et al., 2020) and, (3) with the development of functional potential assessment techniques, an indication of which groups of genes - linked to which metabolic pathways - are associated with each taxonomic group and each set of transformations throughout the fermentation process (Almeida et al., 2020).

Much remains still to be unraveled; however, including information about which features determine the predominance of one taxonomic group over another throughout the fermentation period. In order to deeply investigate functional traits still unexplored and associated with cocoa fermentation, the current study applied shotgun metagenomics (using short-read NGS platform) to determine the enzyme-coding genes of the microbiota present in two different groups of cocoa beans varieties during different fermentation times (between 0 and 144 h). After normalizing their relative abundance, the enzyme-coding genes that were highlighted as significantly more abundant at each time of fermentation were selected, and their potential functionalities were analyzed. Therefore, it was possible to evaluate which groups of enzyme-coding genes were prominent according to the fermented cocoa beans variety and fermentation time, shedding light on the skill sets that ensure the permanence and success of the spontaneous microbiota.

## Materials and methods

### Cocoa samples and analyses

Samples of cocoa (*Theobroma cacao* L.) beans of Forastero variety (FOR) and a mixture of two hybrids (PS1319 and CCN51; MIX) of the fermentation process from the Riachuelo Agroindustry of Mendoá Chocolates^1^ (Uruçuca, Bahia, Brazil; Lat-14.7719058; Long-39.0492701) were analyzed. On this farm, all the tree-to-bar processes (from cocoa planting to the chocolate bar) are done. Furthermore, the Good Manufacturing Practices (GMP) standards in the chocolate agroindustry production, and also encompassing the cocoa planting, management, harvesting, and fermentation process are practiced. Thus, both cocoa beans and chocolate have superior quality and are called fine or gourmet chocolate. Pods were washed, dried, opened, and immediately transferred to wooden boxes (45 × 45 × 45 cm, and capacity of 40 kg).

### Field experimental design

Two blocks (**FOR** and **MIX**) with three fermentation boxes in each one were sampled at seven distinct times: 0 (cocoa beans just after opening the pods), 24, 48, 72, 96, 120, and 144 h. The first spin was carried out after 48 h of fermentation, followed by spins every 24 h, ending on the seventh day of fermentation. Subsamples of 30 g were collected at five random points in the fermentation mass of each wooden box. Subsequently, mixed (total of 150 g sample), and part of it (100 g) was stored at - 20°C for shotgun metagenomics, and part (50 g) was refrigerated (4°C) for physicochemical analyses. The methodology used to Field experimental design was the same as Lima et al. (2020).

### Metagenomic DNA extraction

From the lyophilized pulp fraction of the cocoa beans collected from the wooden boxes, the metagenomic DNA of the samples was extracted according to Cota-Sanchez et al. (2006), and with some modifications, as described by Lima et al. (2020).

An optimized protocol was used based on Cota-Sanchez et al. (2006) with some modifications: 20 g of cocoa mass (pulp + seeds) were placed in sterile glassware with 20 ml of sterilized ultrapure water and vigorously homogenized on a magnetic stirrer for 5 min. Pulp fraction was recovered by decantation and then lyophilized (LP3, Jouan). A total of 0.5 ml of lyophilized with 1 ml of sorbitol buffer 100 mm Tris base, 100 mm Sorbitol, 5 mm EDTA, 2% β-mercaptoethanol and 1% polyvinylpyrrolidone (PVP-40) were placed in a 2.0-ml microtube and centrifuged (Eppendorf 5804R) at 3000 *g* for 10 min. Supernatant was discarded, and liquid nitrogen was introduced to macerate the pellet with sterile rod. This procedure was repeated with sorbitol buffer until the presence of mucilage was no longer observed.

After the washing step, 500 μl of CTAB (100 mm Tris–HCL, 20 mm EDTA, pH 8.0, 2% CTAB, 1.5 NaCl, 4% PVP-40 and 10 mm 2-β-mercaptoethanol), 800 μl STE buffer (100 mm Tris–HCL, 50 mm EDTA, pH 8.0, 100 mm NaCl, 10 mm β-mercaptoethanol), 50 μl 20% SDS (sodium dodecyl sulfate), 10 μl RNAse, 20 μl proteinase K, 50 mg.ml^−1^ lysozyme were manually homogenized (inverting the tube) for 7 min. Subsequently it was incubated in water bath at 65°C for 30 min., with homogenizations at 5-min intervals. Afterwards, it was removed from the water bath, adding 415 μl 3 M ice-cold potassium acetate and incubated on ice for 40 min. Later, it was centrifuged at 25,805 *g* for 20 min. The same volume of ice-cold isopropanol (1: 1) was added, and gently homogenized and incubated at-20°C for 40 min. After, it was centrifuged at 25,805 *g* for 20 min and supernatant was discarded. Pellet was dried at room temperature, resuspended in 500 μl TE (100 mM Tris, 1 mM EDTA buffer pH 8.0) plus 500 μl of chloroform-isoamyl alcohol (24:1), gently mixed and centrifuged at 10,000 rpm for 10 min. Supernatant was transferred to a 1.5-ml microtube and 65 μl of 3 M sodium acetate and 600 μl of ice-cold isopropanol were added, gently mixed, and incubated overnight at-20°C. After that, it was centrifuged at 25,805 g for 20 min, and the supernatant was discarded. Pellet was washed with 1 ml ice-cold ethanol (96%) and centrifuged at 25,805 *g* for 5 min. Supernatant was discarded, and pellet was washed again with 1 ml ice-cold ethanol (75%) and centrifuged at 25,805 *g* for 5 min. Metagenomic DNA was resuspended in 40 μl ultrapure water and stored at-20°C until shotgun metagenomics sequencing.

### Massively parallel sequencing

The metagenomic libraries were prepared according to Illumina’s standard protocols, and the sequencing was performed on an Illumina Hiseq X Ten (Novogene Sequencing Laboratory, UC Davis Medical Center). One μg of DNA per sample was used for sequencing, and the raw sequences were deposited in NCBI SRA under Bioproject accession PRJNA552479.

A total amount of 1 μg of DNA per sample was used for the preparation of metagenomic libraries. Libraries were constructed using DNA NEBNext^®^ Ultra II kit for insertions of 350 bp. The libraries were analyzed by fragment size using Bioanalyzer Agilent 2,100 and quantified by real time PCR (3 nM). Sample clusters codified by indices were carried out in a cluster generation system cBot according to manufacturer instructions. After cluster generation, sequencing was performed on an Illumina Hiseq X Ten (Novogene Sequencing Laboratory, UC Davis Medical Center), generating 150 bp paired end reads. All the raw sequences were deposited NCBI SRA with Bioproject accession PRJNA552479 (SRR9640346, SRR9640343, SRR9640356, SRR9640355, SRR9640354, SRR9640353, SRR9640352, SRR9640351, SRR9640345, SRR9640344, SRR9640348, SRR9640347, SRR9640350, SRR9640349).

### Bioinformatics analysis

For preprocessing the data, we firstly used FASTQC v. 0.11.4 (Andrews, 2010) for quality analysis; CUTADAPT v. 1.18 (Martin, 2011) for adapter removal; BOWTIE2 (v. 2.3.4.3; Langmead and Salzberg, 2012) for Alignment in order to filter for pairs of reads not mapping on *Theobroma cacao* genome (NCBI Criollo cocoa genome V2); and, finally, FLASH v. 1.2.11 (Magoc and Salzberg, 2011) for merging filtered reads. For the taxonomic assignment, the strategies used were the same as in Lima et al. (2020).

Data pre-processing was as follows: (1) FASTQC (v0.11.4; Andrews, 2010) for quality analysis; (2) Adapter removal with CUTADAPT (v. 1.18; Martin, 2011); (3) Alignment using BOWTIE2 (v. 2.3.4.3; Langmead and Salzberg, 2012) to filter for pairs of reads not mapping on *Theobroma cacao* (genome from NCBI; Criollo cocoa genome V2); (4) Filtered reads were merged using FLASH (v1.2.11; Magoc and Salzberg, 2011).

For taxonomic assignment, two strategies were used: (a) the first one using all reads against a database, and (b) another using only the 16S and 18S rDNA reads. The first taxonomic analysis was performed by CENTRIFUGUE (v. 1.0.3; Kim et al., 2016) and KAIJU (v. 1.6.2; Menzel et al., 2016) and used a database composed of genomes selected from RefSeq and Genbank according to the following rules: (i) the genome was not excluded from RefSeq, except if it is from single cell, (ii) it is the latest version, (iii) only one genome per taxid, (iv) bacterial genomes were downloaded from RefSeq, (v) archaeal, fungal and viral genomes were downloaded from Genbank. The following rules were used for maintaining bacterial, fungal, and archaeal genomes: (i) genome was obtained from single cell, or (ii) it is a reference or representative genome, or (iii) it is related to type material, or (iv) it is a complete genome; (v) all viral genomes were downloaded from Genbank.

The genomes were download in September 2018 (Bacteria: 12,361; Archaea: 544; Fungi: 1,536; Virus: 14,132), and two indexes were generated, one for CENTRIFUGUE and another for KAIJU. The index for KAIJU used the same genomes as that of CENTRIFUGUE but, as KAIJU uses amino acids instead of nucleotides, the reads were translated in their six frames. Furthermore, due to RAM limitations, amino-acids sequences equal or less than 30 aa were discarded for KAIJU indexing. Reads were classified using both programs, and the results were merged, giving priority to CENTRIFUGUE results. The second taxonomic analysis was based on the 16S and 18S ribosomal subunits. Using the reads merged by FLASH, the ribosomal subunits sequences were extracted, classified, and mapped in OTUs using MAPSEQ (v. 1.2.3; Matias Rodrigues et al., 2017). The classification results of MAPSEQ were filtered using a combined score above 0.4 as recommended by the MAPSEQ authors. OTUs classified as metazoans or plants were removed.

### Functional prediction and quantification

For the assembly, after the previous processing of the reads, they were trimmed using Trimmomatic (v0.38; Bolger et al., 2014) with parameters “*LEADING:15 TRAILING:15 SLIDINGWINDOW:5:15 MINLEN:50*.” To further reduce the complexity of the metagenome, each sample had their reads binned by their assigned phylum, with the bins of each FOR samples merged with their respective bins and the same for the bins of MIX libraries. Then, each bin was assembled using Spades (v3.13.1; Nurk et al., 2017) with the parameter “*--meta*.” For the quantification, Salmon (v.0.9.1; Patro et al., 2017) was used to generate an index with a merged file using all assemblies, and the contigs were quantified using the “*--meta*” parameter, to change the behavior of the software to quantify metagenomic reads, with the reads without adapters and before trimming. The TPM (Transcripts Per Million) value that is given by Salmon, which is normalized by the length of the contig, and the library size will be called here as CPM (Contigs Per Million) to avoid confusion as these are the values for each genomic contig.

The contigs from procaryote reads had their proteins predicted using Prodigal (v2.6.3; Hyatt et al., 2010) with the parameter “*-p meta*.” For the eukaryote contigs, their proteins were predicted using Augustus (v3.3.3; Stanke et al., 2008) with the parameter “*--species = generic*.” For functional annotation of the predicted proteins, the software Interproscan (v5.44–79; Jones et al., 2014) was used to predict metabolic pathways. Then, the proteins that were annotated with an EC number had the CPM from their respective contigs used as their quantification. For each sample, when there were multiple identical EC numbers from the same protein, they were counted as one, if there were different genes with the same EC number, then they were summed. All the complete scripts are in the Supplementary material 1.

### Statistical analyses

In order to examine variation in enzyme-coding genes composition for each sample of **FOR** and **MIX** samples, we first used a Principal Component Analysis (PCA), transforming input variables to zero mean and unit variance (Husson et al., 2017). Then we performed non-metric multidimensional scaling (NMDS) of individual samples, using Hellinger transformation and the Bray- Curtis; dissimilarity index (Legendre and Gallagher, 2001). We first visually examined the extent to which there was a structure in the ordination, and then we used PERMANOVA (Anderson, 2001) to test for significant clustering with respect to different varieties and fermentation times, using 999. We finally wanted to check whether multivariate dispersion could be different between varieties using PERMDISP (Anderson, 2001). All the analyses were performed in R (R Core Team, 2020), using the packages *FactoMineR*, *Vegan*, and *ggplot2*, and the complete script is in the Supplementary material 2.

The classification of the enzyme-coding genes associated with the main metabolic pathways (lipid, protein, carbohydrate, nucleotide, and micromolecule metabolisms) was based on enzyme EC and corresponding KEGG id using the website.^2^ The variation of the abundance of enzyme-coding genes along the fermentation time was analyzed and visualized using color matrices. The results were compared over time [T1 (24–48 h) × T2 (72–96 h) and T2 × T3 (120–144 h)] and were considered significant when a value of p less than 0.05 and a fold change higher than 2.5 were found. Unique enzyme-coding genes have been present at all times; however, in very low abundances. Only the statistically significant shared enzyme-coding genes were then analyzed.

## Results

**Figure 1:**
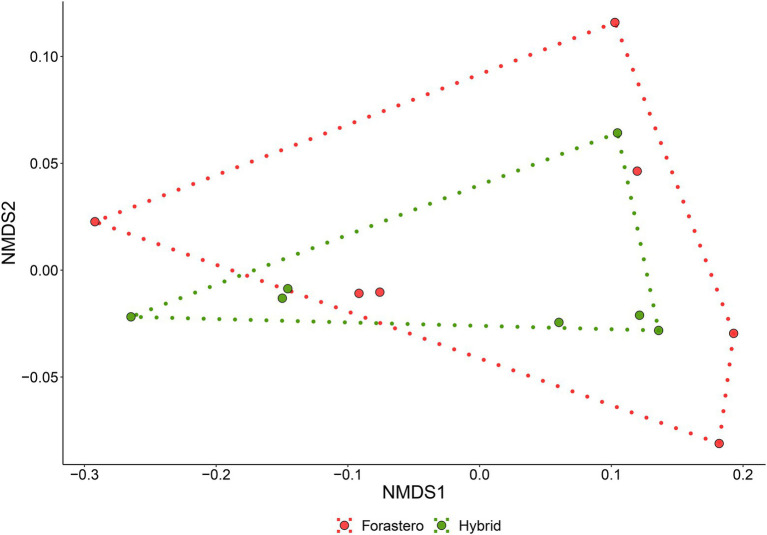
Permutational Dispersion plot of cocoa varieties samples in distinct fermentation times. Black circles for FOR (Forastero) and red triangles for MIX (mixture of two cocoa beans hybrid varieties).

**Figure 2:**
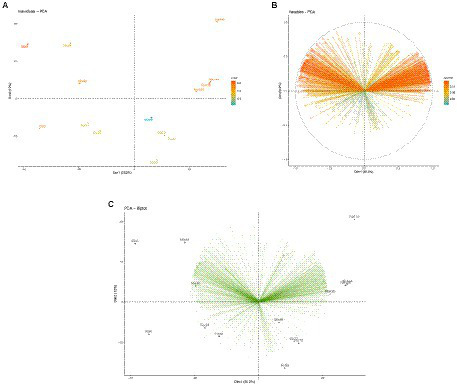
**(A)** PCA plot showing only the cocoa beans varieties samples in distinct fermentation times; **(B)** PCA plot showing only the enzyme-coding genes; **(C)** PCA biplot showing both the samples and enzyme-coding genes.

The non-metric multidimensional scaling (NMDS; Supplementary material 3) jointly with a permutational dispersion (PERMDISP) test (Figure 1) clearly indicated that (except for the T0 samples, which were not evaluated hereafter) the multivariate dispersion of the two cocoa beans varieties (FOR and MIX) was not significantly different, and, thus, they can be treated as duplicates of one same entity. Furthermore, the Principal Component Analysis (PCA; Figures 2A–C) showed a clearly distinct ordination of both varieties of cocoa beans on different fermentation times 24-48 h, 72-96 h, and 120-144 h, which were hereafter named as T1, T2, and T3, respectively.

Most of the shared statistically significantly enzyme-coding genes in each T1, T2, and T3 times were directly associated with the dominant core microbiome fungal genus *Hanseniaspora* (T1) and bacterial genus *Acetobacter* (T2). *Hanseniaspora* is described as one of the most common fungal genus, besides *Saccharomyces* and *Pichia*, as revealed by both culture-dependent and culture-independent studies (Arana-Sanchez et al., 2015; De Vuyst and Weckx, 2016; Schwenninger et al., 2016; Koffi et al., 2017; Pereira et al., 2017; Mota-Gutierrez et al., 2018; Papalexandratou et al., 2019; Lima et al., 2020). *Acetobacter* has already been documented in cocoa fermentation, in different regions and by different methods (Camu et al., 2008; Garcia-armisen et al., 2010; Papalexandratou et al., 2011c; Pereira et al., 2012, 2013; Crafack et al., 2013; Ramos et al., 2014; Hamdouche et al., 2015; Schwenninger et al., 2016; Visintin et al., 2016; Moreira et al., 2017; Gabaza et al., 2019; Lima et al., 2020). These data suggest that *Acetobacter* is also a core bacterial genus in cocoa bean fermentation worldwide.

Moreover, these aforementioned enzyme-coding genes were also related to other relatively abundant bacteria or fungi, such as *Pantoea, Lactobacillus, Frateuria, Candida,* and *Rhizopus* in T1; *Komagataeibacter*, *Frauteria*, *Gluconobacter*, *Pantoea,* and *Sphingomonas* in T2; and *Komagataeibacter* in T3 (Supplementary material 4).

**Figure 3:**
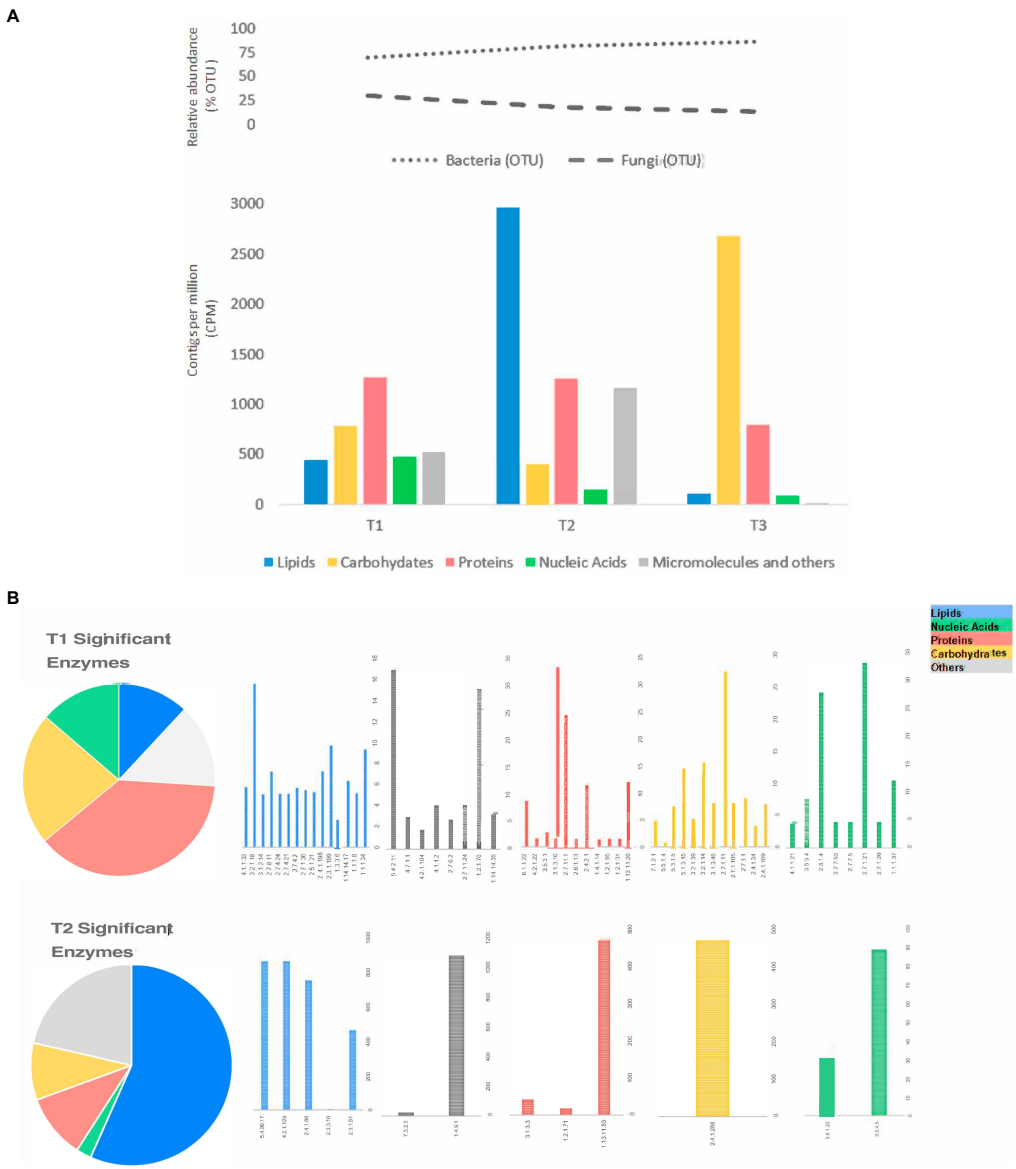
**(A)** Relative abundance of bacteria and fungi (%). **(B)** CPMs of potential exclusive or dominant enzymes related to essential metabolic pathways during cocoa fermentation: T1 (24 - 48h), T2 (72 - 96h), and T3 (120 - 144h).

Regarding the genes encoding enzymes, there was a greater abundance for those related to the metabolism of proteins and carbohydrates within 48 h of fermentation. This coincides with the presence of a high concentration of bacteria and yeasts present in the fermentation medium whose results were previously found by our research group and reported in Lima et al. (2020). The abundance of genes related to lipids metabolism is evident in T2 followed by genes related to carbohydrate metabolism in T3; this set of genes could be related to the stress response to which the microorganisms are subjected since it coincides with changes in the environment in relation to pH and temperature that occur during the fermentation process of almonds (Figure 3).

**Figure 4:**
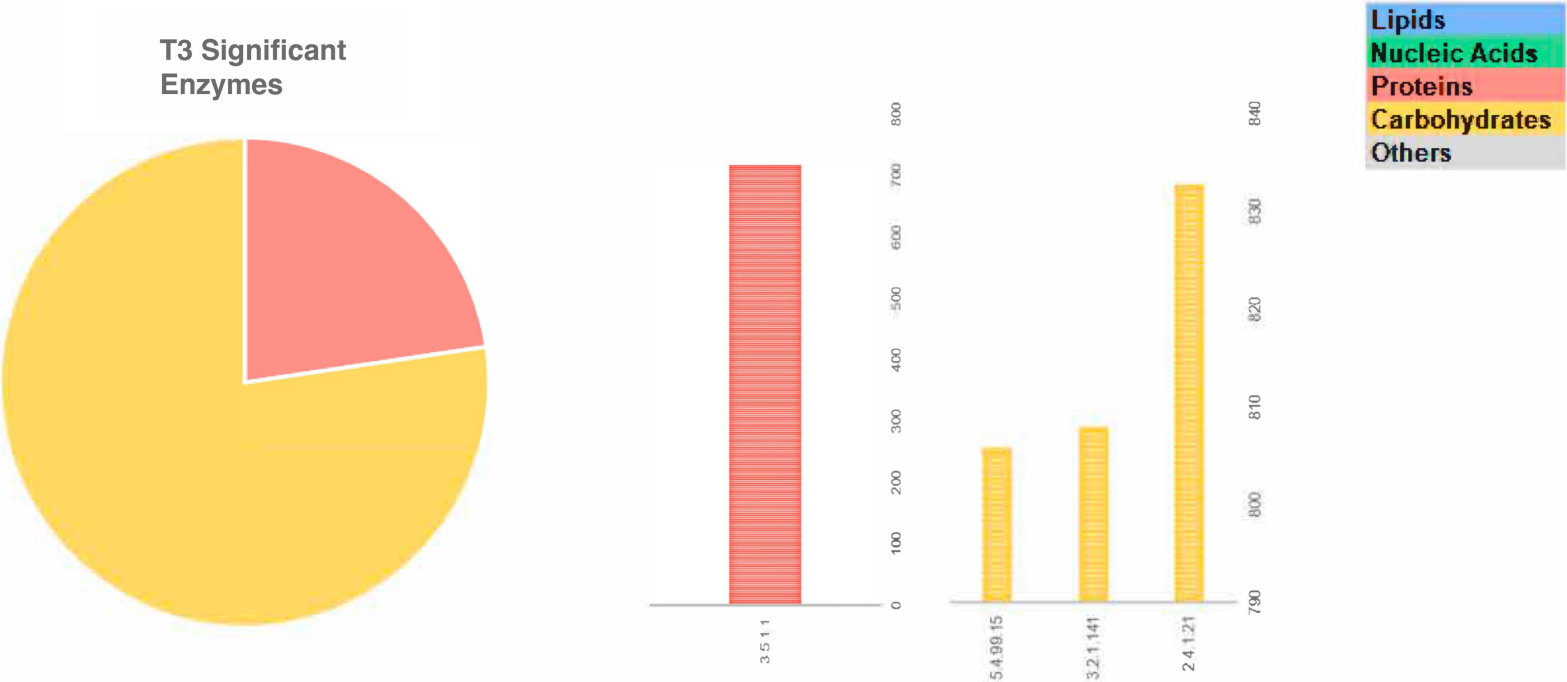
Significant enzyme-coding genes with greater abundance at different fermentation points (a – T1 and T2; b – T3), which were involved in the metabolism of lipids, carbohydrates, proteins, nucleic acids, micromolecules and others in fine cocoa beans fermentation.

Among the significant genes with greater abundance at different fermentation times are those involved in the metabolism of proteins and carbohydrates in T1, followed by the genes involved in the metabolism of lipids in T2 and carbohydrates in T3 stand out (Figure 4).

In general, at T1, a higher number of genes was found significantly more abundant, for all five categories evaluated (metabolism of lipids, carbohydrates, proteins, and amino acids, nucleic acids, and others). The categories with the highest number of significantly more abundant genes were ‘lipid metabolism’ and ‘carbohydrate metabolism’, with 15 representatives each, although the ‘lipid related’ genes accounted for just about 12% of the total abundance where ‘carbohydrate related’ genes reached almost 30%.

About lipid metabolism, of the 15 highlighted lipid-related genes at T1, 4 encode enzymes of the mevalonate pathway for the synthesis of isoprenoid precursors, such as farnesyl (1.1.1.34; 2.7.1.36; 4.1.1.33; 2.7.4.2), together representing 26% of the abundance of this category; 2 encode enzymes in the biosynthesis of steroids, specifically those that transform farnesyl into squalene and this into its epoxide (2.5.1.21; 1.14.14.17–11.5% of the abundance); 3 encode enzymes in the metabolism of glycerophospholipid, related to the myoinositol phosphate metabolism pathway (1.1.1.8; 2.4.1.198; 2.7.8.11–19.5% of the abundance); and 2 are involved in the phosphatidylinositol signaling system (2.7.4.21; 2.7.4.24–10% of the abundance). One gene encodes an enzyme for the synthesis of fatty acids (3.1.2.14) with the possibility of leading to the elongation of fatty acids or the metabolism of glycerophospholipids; 1 encodes an enzyme from the fatty acid elongation pathway (2.3.1.199), 1 encodes an enzyme of the fatty acid beta- oxidation/degradation pathway (1.3.3.6) and 1 encodes an enzyme in the sphingolipid metabolism (3.2.1.18). The latter gene was the most abundant in this category, alone representing about 15% of the total abundance of genes encoding enzymes of the metabolism of lipids. At T2, the number of significantly more abundant genes dropped to just 13. Of these, five were related to lipid metabolism, which stood out in this stage of fermentation, corresponding to more than 58% of the total abundance found. Among the five highlighted genes, two stood out, with about 30% of the total abundance, each: both encoding enzymes of the metabolism of hopanoids – pentacyclic triterpenes (5.4.99.17 and 4.2.11.22), one encodes a glycosylceramide synthesizing enzyme in the sphingolipid metabolism (2.4.1.80–25% of the abundance), one encodes an enzyme in the metabolism of glycerophospholipid (2.3.1.51–15.65% of the abundance) and one, with much lower abundance than the others (2.3.3.10–0.13% of the abundance), encodes a hydroxy-methyl-glutaryl-CoA synthase, involved in several metabolic pathways, including the formation of the carbon skeleton for terpenoids.

Thus, despite lipid metabolism having not been directly related to the generation of chocolate flavor in cocoa fermentation to date, there are strong indications that it is essential for the survival of strains in the hostile environment that is installed along the process, which indirectly influence flavor formation.

Regarding carbohydrate metabolism, in T1, of the genes involved in carbohydrate metabolism, most are those involved in the glycolytic pathway such as 6-phosphofructokinase (2.7.1.11–20.5% of the abundance) and hexokinase (2.7.1.1–5.7%) and H + −exporting transporter (7.1.2.1–3%) involved in ADP phosphorylation in phosphorylation oxidative. The malate dehydrogenase gene (1.1.1.37) that catalyzes the conversion of malate to oxaloacetate in the tricarboxylic acid cycle (TCA) was found with an abundance of 5.75%. Regulatory genes for glycolysis and gluconeogenesis (6-phosphofructo-2-kinase, 2.7.1.105–5.3%) as well as those involved in the metabolism pathway of pentoses fructose-2,6-bisphosphate 2-phosphatase (3.1.3.46–5.3%), phosphoglycerate mutase (5.4.2.11–15.25%), and glucose-6-phosphate 1-epimerase also are present (5.1.3.15–9.3%). The oxalate decarboxylase is involved in glyoxylate metabolism in the conversion of oxalate to formate (4.1.1.2–3.73%). Genes involved in chitin metabolism (3.2.1.14–10%) were also found and genes involved in the cell wall metabolism of microorganisms such as dolichyl-phosphate-mannose-protein (2.4.1.109–5%), inositol-3-phosphate synthase (5.5.1.4–0.5%), and 1, 3-beta-glucan synthase (2.4.1.34–2.4%). Genes involved in the interconversion of aldose and ketose sugars and in the metabolism of glycogen also appear (xylose isomerase - 5.3.1.5 and glucan endo-1,3-beta-D-glucosidase - 3.2.1.39) with 4.9 and 3.4% of the abundance, respectively.

In T2 the gene which encodes to arabinogalactan biosynthesis of peptidoglycan in mycobacterium is predominantly present (2.4.1.288). Nonetheless, at T3 the abundance of the gene could be involved in the trehalose disaccharides pathway (3.2.1.141, 5.4.99.15—both with 33% of the abundance each) and glycogen production (2.4.1.21–34%).

About protein and amino acid metabolism, considering the abundance of all the 11 genes present in fermentation at T1 related to amino acids and protein metabolism, the genes involved in serine/threonine dephosphorylation (3.1.3.16) and phosphorylation (2.7.11.1) are the most abundant, with 36 and 27%, respectively. The genes involved in the cysteine metabolic pathway (1.3.11.20, 4.2.1.22) account for 15% while for arginine and proline metabolism (2.6.1.13, 3.5.3.1) was 4.8%, lysine (1.2.1.31, 1.2.1.95) was 3.3% and glutamate (1.4.1.14) just 1.5% of abundance. The mitogen-activated protein kinase (MAPK; 2.7.11.24) signal transduction pathways, which are among the most widespread mechanisms of cellular regulation, and asparagine-tRNA ligase (6.1.1.22) were also present with 3.2 and 9.2% of abundance, respectively. Among the total of genes present at T2, 11% of them correspond to genes related to the metabolism of proteins and amino acids and, of these, 88.9% abundance was found for the metabolism of cysteine and methionine (1.13.11.53) in the conversion of the metylterpene in methylpropionate and less abundance to arginine (1.2.1.71–3.5%) and serine (3.1.3.3–7.9%) pathway. However, at T3, of the total gene, 1 was to the proteins and amino acid metabolic pathway, and the asparagine gene (3.5.1.1–23% of abundance) involved in the conversion of L-asparagine to aspartate was found.

At T1, when compared to T2, genes encoding enzymes involved in nucleic acid metabolism accounted for 12.2% of the significantly more abundant genes. Six genes encoding enzymes related to the metabolism of purines and pyrimidines (2.7.1.21; 2.4.2.1; 3.5.3.4; 2.7.7.53; 2.7.1.20; 4.1.1.21). At T2, only one gene encoding an enzyme of the metabolism of nucleic acids was highly abundant: 3.5.4.5 - related to the metabolism of pyrimidines. The representativeness of this group of genes at T2 dropped notably, to about 1.8% of the total of the significantly more abundant genes, indicating the deceleration in DNA/microorganism duplication throughout fermentation. In addition to genes related to encoding metabolism enzymes from the four main groups of biomolecules, significantly more abundant genes encoding enzymes from other metabolic groups were also found. At T1, this group of genes represented about 12% of the genes considered. Among the genes in this group, participants in the metabolism of B-complex vitamins (2.8.1.4; 2.7.6.2 – thiamine and 6.3.1.20 – lipoic acid – 43% of the total), of porphyrins (1.2.1.70–35% of the total), of sulfur metabolism (1.14.14.35; 2.7.7.5–13.45% of the total), phosphorus (4.7.1.1) and nitrogen (4.2.1.104) were found. At T2, the genes of this group got to represent almost 23% of the highlighted genes abundance, the second most represented group, and almost 95% of the abundance was due to the gene encoding the enzyme methylamine dehydrogenase (1.4.9.1) of the metabolism of methane, although genes encoding enzymes of nicotinic acid and sulfur metabolism were also highlighted.

## Discussion

Based on the genes encoding enzymes, there was a greater abundance for those related to the metabolism of proteins and carbohydrates after 48 h of fermentation, with the greater presence of genes involved with the metabolism of fructose and glucose. These results are probably due to the abundance of bacteria of the genus *Gluconobacter* present in the cocoa fermentation, where they were the most abundant in the 0 h time. In addition, this bacterial genus was present in a relatively high abundance up to 48 h of fermentation (T1), being after that time overlapped by the most abundant presence of bacteria of the genus *Acetobacter* (Lima et al., 2020). The abundant presence of yeast of the genus *Hanseniospora* throughout the fermentation process also explains an abundance of genes related to the metabolism of carbohydrates at T1 and T3. According to Gálvez et al. (2007), *Hanseniaspora* is associated with the intense metabolism of sugars, generating ethanol, and exhibiting pectinolytic activity.

During cocoa beans fermentation, amino acids are precursors to produce different flavor compounds (Spinnler, 2012). We found genes coding for serine and threonine at T1 with greater abundance (63%) and at T2 (7.9%), these would be involved in the intermediary metabolic pathway to produce the amino acid valine, which is a precursor of saturated fatty acids. Studies withthe genome of *Saccharomyces cerevisiae*, *Bacillus subtilis* subsp. subtle str. 168, *Limosilactobacillus fermentum* IFO 3956 and *Acetobacter aceti* were used to build a metabolic pathway for L-leucine, L-phenylalanine, L-tyrosine. L-tryptophan, valine, and L-threonine showing that these amino acids are crucial as flavor and odor precursors and bioactive compounds in fine cocoa (Fernández-Niño et al., 2021).

The methionine may be involved in sulfur aroma compounds; these metabolites are also present in other fermentation processes such as in the production of cheese, wine, and fruit ripening (Spinnler, 2012). In our study, the presence of this gene was found in T2 (89% of the abundance) as well as cysteine, arginine, and serine, while at T3 the asparaginase represented the total of genes found for this metabolism. Similar to our results, the presence of some amino acid genes involved in flavor and aroma were found by Illeghems et al. (2015) that showed it could be associated with lactic acid bacteria (LAB) during the cocoa bean fermentation process.

Analyses carried out in other studies during cocoa fermentation showed that the peak concentration of peptides and amino acids is related to the increase in fermentation days (3–4 days) and the metabolic changes during fermentation associated with proteolysis, such as the production of peptides, amino acids, and polyphenols, are important to the formation of the typical flavor and aroma during the roasting of cocoa beans (Mayorga-Gross et al., 2016; Herrera-Rocha et al., 2021). Similarly, in the study, the abundance of protein-related genes was found at the beginning of fermentation, at T1 (24 - 48 h), which could be related to proteolysis and release of amino acids and peptides. Nevertheless, these compounds were not analyzed, and additional experiments would be necessary to elucidate this hypothesis in the cocoa beans fermentation process.

The presence of many genes involved in carbohydrate metabolism was expected since at the beginning of the fermentation process there is a great availability of this compound. The cocoa pulp is rich in simple carbohydrates (glucose and fructose), and sucrose (Verse Herrera-Rocha et al., 2021). The fact that many genes are presently involved in different pathways for the metabolization of carbohydrates is corroborated by the work described by Pothakos et al. (2020) in the Ecuadorian coffee fermentation process. The authors also verified several enzyme genes related to glycolysis and pentose-phosphate pathways which would be favoring the release of intermediates for the formation of lactic acid or acetic acid by the bacteria present at this time of fermentation. Besides that, the acetyl-CoA produced could be converted into ethanol. The production and consumption of ethanol are reflected in the production of acetic acid by AAB (Verce et al., 2021), which explains the abundance of *Acetobacter* bacteria in the 24 h of fermentation and remains until the end of the fermentation (Lima et al., 2020).

The abundance of genes encoding enzymes that are involved in the production of secondary metabolites was evident in T1 probably due to a response to the stress of the medium that was modified mainly by the action of AAB bacteria after 24 h of fermentation, with consequent acidification of the medium and increased temperature (Lima et al., 2020). There are also reports in the literature relating the tolerance of AAB to the constitution of phospholipids, glycolipids, and carotenoids such as tetrahydroxybacteriohopane (THBH) in their cell membrane (Qiu et al., 2021).

The presence of the squalene-hopene cyclase gene is related to the formation of THBH in AAB cell membranes and this gene is abundantly present in T1 and T2 in the present work, which could confirm the involvement of THBH in resistance by AAB in the presence of high concentrations of acetic acid. In addition, the resistance of these bacteria to high levels of acetic acid is also due to the maintenance of low levels of intracellular acetic acid that occurs through the ABC transporter (Nakano et al., 2006; Qiu et al., 2021).

The abundant presence of yeast of the genus *Hanseniaspora* throughout the fermentation process (Lima et al., 2020) also explains an abundance of genes related to the metabolism of carbohydrates in T1 and T3. Studies indicate that there could be changes in the yeast cell wall related to fermentative processes that are halotolerant or osmotolerant, where greater flexibility of the cell wall, related to the presence of mannans, could be involved in the greater resistance of these microorganisms to these extreme environments compared to the microorganisms that have more rigid cell walls, which tend to be less halotolerant (Dakal et al., 2014). Thus, the abundance of genes “dolichyl-phosphate-mannose-protein” and “1,3-beta-glucan synthase” found in T1 could be involved in the modulation of β-D-glucan and cell wall mannans yeast synthesis which can undergo changes in responses to osmotic stress, considering the presence of high concentrations of sugar in the medium in the initial fermentation times.

Additionally, the presence of the abundance of genes (1- > 4)-alpha-D-glucan 1-alpha-D- glucosylmutase and 4-alpha-D-{(1- > 4)-alpha-D-glucan} trehalose trehalohydrolase that catalyzes the trehalose synthesis in T3, could be related to the stress response to which microorganisms were exposed, mainly to osmotic and thermal stress. The presence of trehalose has been described as involved in bacterial and yeast responses to different environmental stress, such as resistance to osmotic pressure or in relation to extreme temperatures (De Virgilio et al., 1994; Iturriaga et al., 2009). Experiments carried out by Reina-Bueno et al. (2012) with *Chromohalobacter salexigens* mutants showed that trehalose synthesis is regulated by osmotic stress at the transcriptional level of the trehalose-6- phosphate synthase gene and in heat stress there is a post-transcriptional regulation, leading to an osmotic and thermoprotection. In general, it can be stated that the genes related to lipid metabolism, significantly more abundant at T1, are related to the synthesis of squalene, glycerophospholipids, and sphingolipids. According to Lima et al. (2020), after T1 (24-48h) the microbial richness, evenness, and Shannon diversity drastically decrease, indicating the reduction in the number and diversity of the microorganisms in this process. At T2 the significantly more abundant genes are related to the synthesis of glucosyl-ceramide and hopanoids, the latter straight derived from squalene (Belin et al., 2018). All these products can be directly related to microbial resistance to hostile environments, whether due to changes in pH or to elevated temperatures.

Hopanoids are branched cyclic triterpene compounds derived from squalene, which are similar to sterols. Similarly, to these compounds, hopanoids can associate with other lipids in the cell membrane of prokaryotes, altering their fluidity and permeability. Hopanoids concentration in the cell membrane of bacteria can vary from less than 1% to more than 90%, in peripheral, intra-, and extracellular membranes, depending on the species and on the environmental conditions, and their presence leads to the condensation, thickening, and reduction of permeability of these membranes. Not only the concentration of hopanoids but also the type and the occurrence of interactions with sphingolipids, forming the so-called ‘lipid rafts’, interfere in the properties of the membranes, promoting resistance without significant loss of fluidity. Hopanoids are related to the survival of bacteria under stressful conditions. Their concentration rises at high temperatures and in the presence of acidic pH, such as found during cocoa fermentation. Apparently, the presence of hopanoids in membranes reduces the loss of cations and protons to the acidic environment, as well as protects the membrane structure against thermolysis in a heated environment (Belin et al., 2018; Santana-Molina et al., 2020).

Bacteria and fungi also use sphingolipids and ceramides to modulate the melting point of cell membranes to overcome high temperatures. Sphingolipids show a higher melting point than membrane phospholipids and their composition may be changed according to the temperature of the environment to ensure fluidity and permeability of cell membranes. In yeasts, the acquisition of thermotolerance depends on the fast accumulation of ceramides and sphingolipids such as glucosyl- ceramide (although this is not produced by *S. cerevisiae*) and glycosyl-inositol-phosphoryl-ceramide. The accumulation of these products is related to the long-term thermal resistance in fungi (Fabri et al., 2020).

Our results seem to disagree with the data published by Herrera-Rocha et al. (2022) and also by Servent et al. (2018). In the former, the authors concluded that there is no change in the content of lipids during fermentation, including glycerophospholipids, the second most abundant group of lipids in fermenting cocoa. In the latter, the authors found evidence of the release of fatty acids, possibly by lipases - not found in the present study - but did not evidence change in the total lipid content, nor did they mention the presence of glycerophospholipids. However, Servent et al. (2018) made it clear that the behavior of lipids in cocoa was dependent on the fermentation origin, as demonstrated by PCA. This leads us to believe that similar to other transformations that occur during cocoa fermentation, the changes in the lipid fraction are directly dependent on the composition of the microbiota involved and indicates that, in the case of the present study, some change in the composition of glycerophospholipids is to be expected. As there are no details on the fermentative microbiota in Herrera-Rocha et al. (2022), nor in Herrera-Rocha et al. (2021) nor in Servent et al. (2018), the proof of this hypothesis will depend on additional experiments.

The results obtained indicate that the genomic machinery involved in resistance to hostile environments, such as acidic pH and high temperature, is essential for the microbiota of cocoa fermentation, regardless of the cocoa varieties used as substrate and of the initial fermentation microbiota, providing a significant advantage to those resistant species and ensuring their survival in this specific process/environment.

The *de novo* synthesis of purine and pyrimidine bases is essential for cell multiplication, which may explain the high representation of genes related to this function in the initial stages of fermentation—the metabolism of nucleic acids at T1 stood out as the 3rd largest group of genes among those significantly more abundant, only behind the metabolism of carbohydrates and proteins which, according to Almeida et al. (2020), represent the main portion of the functionalities found in the metagenome of cocoa spontaneous fermentation. However, also according to the same authors, it is possible that the metabolism of nucleic acids works as a secondary source of energy, related to the pentose-phosphate pathway.

Among the genes encoding enzymes for the metabolism of biomolecules other than carbohydrates, proteins, lipids, and nucleic acids, those related to the metabolism of B-complex vitamins were remarkable at T1. Two genes, corresponding to 38.6% of the total, 1 encoding an enzyme at the beginning of the thiamine synthesis pathway from cysteine and another encoding an enzyme at the end of this pathway, for the generation of thiamine diphosphate (TPP), were found among the genes significantly more abundant. In addition, 1 gene encoding a ligase from the metabolism of lipoic acid was also evidenced. Thiamine and lipoic acid are important coenzymes of energy metabolism. Their presence is essential for the activity of the pyruvate-dehydrogenase complex and is also necessary for other decarboxylation activities in the metabolism of carbohydrates, amino acids, and fatty acids. According to Agyirifo et al. (2019), yeasts, such as those of the genera *Saccharomyces* and *Hanseniaspora*, found in spontaneous cocoa beans fermentations, can produce large amounts of thiamine, enriching the medium, which can be beneficial for the subsequent development of lactic acid bacteria, especially of the genus *Lactobacillus*, dependent on thiamine from the environment to grow (Carr et al., 2002). It is interesting to note that lipoate synthesis always occurs as bound to proteins, from which it can be separated by specific enzymes (lipoamidases; Cronan et al., 2005). Some organisms can use protein-free lipoic acid through the activity of the enzyme lipoate-protein ligase, of which the encoding gene was found enriched at T1, and that uses ATP to bind lipoate to proteins and re- establish its activity (Cronan et al., 2005). The ability to synthesize this enzyme could mean a competitive advantage for a species to establish itself in an adverse environment.

Glutamyl-tRNA reductase (1.2.1.70), encoded by another enriched gene at T1, makes up the first part of the metabolic pathway for the synthesis of 5-aminolevulinate from glutamate. This metabolite can generate several porphyrins, including protoporphyrin IX, which generates the ‘heme’ group, essential for the formation of cytochromes a and c and various oxidative enzymes, such as peroxidases and catalases, among others. This variety of applications can explain the representativeness of this gene, accounting for more than 1/3 of the total. Verse Herrera-Rocha et al. (2021) related different porphyrin-active proteins (oxygen-dependent coproporphyrinogen-III oxidase; peroxiredoxin) to oxygen-sensing and stress during cocoa fermentation.

The gene encoding the enzyme sulfate adenylyltransferase (2.7.7.5) was also found enriched in T1. This enzyme catalyzes the formation of APS (adenylyl-sulfate) from sulfate captured from the environment. APS is the starting compound for different forms (assimilative and dissimilative) of sulfur metabolism, and also participates in the synthesis of methionine in eukaryotes (*S. cerevisiae*). The other gene encoding an enzyme linked to sulfur metabolism, dimethylsulfone monooxygenase (1.14.14.35), found enriched at T1, generates methane sulfonate, which, in turn, generates sulfite and sulfide, the latter destined for the synthesis of cysteine. To our knowledge, sulfur metabolism has not been mentioned/explored in recent work on cocoa fermentation microbiota.

The gene encoding the enzyme alpha-D-ribose 1-methylphosphonate 5-phosphate C-P- lyase (4.7.1.1) was also found enriched at T1. This enzyme is part of the C-P lyase pathway, which involves an enzyme complex capable of transforming phosphonates (organic molecules containing stable covalent bonds between phosphorus and carbon, which are difficult to lyse and use) into phosphates, that can be used by cell metabolism. Some bacteria have this enzymatic machinery that gives them an advantage in the use of phosphorus from the environment in forms other than soluble phosphate (Manav et al., 2018). Possibly, the presence of these genes provides a competitive advantage for the permanence/predominance of these bacteria in the adverse environment of cocoa fermentation.

The gene encoding cyanase also stood out among those significantly more abundant in T1. The enzyme cyanate lyase (4.2.1.104) degrades cyanate to carbon dioxide and ammonia using bicarbonate as a co-substrate and allows bacteria to use cyanate as the sole source of nitrogen in environments with low availability of this element, also providing a competitive advantage to the microorganisms that establish themselves in the spontaneous fermentation of cocoa (Linder, 2019).

At T2 the gene encoding the amicyanin enzyme (1.4.9.1) was substantially noticed. This enzyme is part of a complex of 3 electron-transporting proteins (methylamine dehydrogenase, blue copper protein amicyanin, and cytochrome c551i) that leads to the deamination of methylamine generating formaldehyde and ammonia (Chen et al., 1994), in the methane cycle. It is possible that this gene was highlighted because of the activity of C-P-lyase (4.7.1.1) at T1 that generates methane.

Also highlighted in T2, although in much less abundance, it was reported the gene encoding the enzyme NAD+ diphosphatase (3.6.1.22). Its activity generates NAD+ and deamino-NAD+ from nicotinamide-D-ribonucleotide and nicotinate-D-ribonucleotide, respectively. These are extremely important coenzymes in general and energy metabolism. According to Agyirifo et al. (2019), yeasts, characteristic of the beginning of cocoa fermentation, are producers of nicotinic acid and nicotinamide, which are the basis for the predominant microorganisms in T2 to synthesize the coenzymes necessary for their metabolism.

The changes/differences in abundance of the genes across T1, T2, and T3 can be stated on changes in microbiome conditions during the period of fermentation. Nutrients and metabolites, pH, temperature could be impacting on species diversity and their metabolism.

Another gene encoding a sulfur metabolism enzyme also stood out among the significantly more abundant genes – the ABC-type sulfate transporter (73.2.3), a transporter protein responsible for the active uptake of sulfate from the environment, feeding the sulfur pathway. Its activity is immediately followed by the activity of the sulfate adenylyltransferase enzyme (2.7.7.5), of which the encoding gene was found enriched at T1, reinforcing the importance of sulfur metabolism for the permanence/predominance of cocoa fermentation microbiota over time.

The results obtained help to better understand the community metabolism of microbial succession throughout the spontaneous fermentation of different varieties of fine cocoa beans unraveling potential enzymes involved in that process, which could improve cocoa processing practices and more stable quality end products.

## Data availability statement

The datasets presented in this study can be found in online repositories. The names of the repository/repositories and accession number(s) can be found in the article/Supplementary material.

## Author contributions

CL: conceptualization, methodology, validation, investigation, writing—original draft, writing—review and editing, and visualization. AV: formal analysis and writing—review and editing. GC: methodology, software, formal analysis, writing—original draft, and writing—review and editing. FL: software, formal analysis, writing—review and editing. RS: methodology, formal analysis, writing—original draft, writing—review and editing. CR: methodology and writing—review and editing. LP: methodology and formal analysis. LV: writing—review and editing and project administration. GP, VA, and RB: writing—review and editing. AC, AU, and MK: conceptualization, methodology, validation, investigation, writing—original draft, writing—review and editing, and visualization. CS: project administration and funding acquisition. AG-N: conceptualization, methodology, validation, investigation, writing—original draft, writing—review and editing, visualization, project administration, and funding acquisition. All authors contributed to the article and approved the submitted version.

## Funding

This work was funded by the Coordination of Superior Level Staff Improvement (CAPES, Grant PROCAD 88881.068458/2014–01). The funder had no role in study design, data collection, analysis, and decision to publish or prepare the manuscript. AG-N receives a research grant for productivity from the National Council for Scientific and Technological Development (CNPq), Brazil (no. 310764/2016–5).

## Conflict of interest

The authors declare that the research was conducted in the absence of any commercial or financial relationships that could be construed as a potential conflict of interest.

## Publisher’s note

All claims expressed in this article are solely those of the authors and do not necessarily represent those of their affiliated organizations, or those of the publisher, the editors and the reviewers. Any product that may be evaluated in this article, or claim that may be made by its manufacturer, is not guaranteed or endorsed by the publisher.

## Footnotes

^1^ www.mendoachocolates.com.br

^2^ https://www.brenda-enzymes.org/
