# Supplementary material for: Unraveling potential enzymes and their functional role in fine cocoa beans fermentation using temporal shotgun metagenomics
Source: Front Microbiol. 2022 Nov 3;13:994524. doi: 10.3389/fmicb.2022.994524 (PMC9671152; doi:10.3389/fmicb.2022.994524)
Supplement: Supplementary file 1 [file Table_1.DOCX]

SUPPLEMENTARY MATERIAL 1: complete scripts for assembly and annotation

# Name: add_contig_name_and_quant.pl

#! /usr/env perl

#AUTHOR:GIOVANNI MARQUES DE CASTRO

use strict; use warnings;

open(ANOT,$ARGV[0]) ||die "Could not open ANOT $ARGV[0] : $! \n"; #"EC_KEGG_interpro_uniq.tsv"

my $file_path=''; my %names;

print "SAMPLE\tTAXA\tFILE\tCONTIG\tPREDICTED\tGO\tKEGG\n"; while(<ANOT>){

my $linha=$_;

$linha=~s/:/\t/;

my @columns=split(/\t/,$linha); # $line[0] is path

$columns[0]=~s/\//\t/g;

if ($columns[1]=~/NODE/){

my $contig=$columns[1];

$contig=~s/_\d+$//;

print "$columns[0]\t$contig\t$columns[1]\t$columns[2]\t$columns[3]";

}else{

if($columns[0] eq $file_path){ print

"$columns[0]\t$names{$columns[1]}\t$columns[1]\t$columns[2]\t$columns[3]";

}else{

%names=();

$file_path=$columns[0]; my $gff=$columns[0];

$gff=~s/\t/\//g;

$gff=~s/Interproscan.tsv/augustus_generic.gff/; open(GFF,"<$gff")|| die "could not open to read gff file: $gff\n"; while($gff = <GFF> ){

if($gff =~/^(\S+)\t.*\ttranscript\t.*\t(\S+)$/){

# my $contig_id=$1;

# my $predicted_gene=$2;

$names{$2}=$1;

# $names{$predicted_gene}=$contig_id;

}

}

print "$columns[0]\t$names{$columns[1]}\t$columns[1]\t$columns[2]\t$columns[3]";

}

}

}

# Name: add_taxaName.pl

#! /usr/bin/env perl

#AUTHOR:GIOVANNI MARQUES DE CASTRO

use Bio::Taxon;

use Bio::Tree::Tree; use Getopt::Long; use warnings;

use strict; my $tax ="";

my $rank='phylum'; my $usage=" USAGE:

perl $0 -tax kaiju.out -rank phylum

-tax : List of NCBI taxids, one per line

-rank: Desired taxonomy rank from the lineage of the taxid (phylum)

";

GetOptions (#"length=i" => \$length, # numeric "taxfile=s"=>\$tax,

"rank=s"=>\$rank #string (defaults to phylum) # "verbose" => \$verbose # flag

)||die "$usage";

open(IN,$tax)||die "No file\n$usage\n"; # Get one from a database

my $dbh = Bio::DB::Taxonomy->new(-source => 'flatfile',

-directory=> '/home/giovannimc/genomas_and_databases/NCBItaxonomy/BioTaxon',

-nodesfile=> '/home/giovannimc/genomas_and_databases/NCBItaxonomy/nodes.dmp',

-namesfile=> '/home/giovannimc/genomas_and_databases/NCBItaxonomy/names.dmp'); my $taxon = $dbh->get_taxon(-taxonid => '9606');

my %taxids;#faster taxon search; my @ranks=

("superkingdom","kingdom","phylum","class","order","family","genus","species");

print "NAME\t".join("\t",@ranks)."\ttaxid\n"; while(<IN>){

my $line=$_; chomp($line);

my @line=split(/\t/,$line);

my $taxid = $line[2]; print "$line[1]"; if(!$taxids{$taxid}){

my $taxon = $dbh->get_taxon(-taxonid => $taxid); if (!$taxon ){

$taxon="\tNA" x 8;

$taxids{$taxid}="$taxon\t$taxid\n"; print "$taxon\t$taxid\n";

next;

}

my $tree = Bio::Tree::Tree->new(-node => $taxon); foreach $rank (@ranks){

my $node = $tree->find_node(-rank => $rank); if($node){ #node has phylum, or any other desired rank

$taxon=$node->scientific_name;

$taxids{$taxid}.="\t$taxon";

}

else{

}

}

$taxids{$taxid}.="\tNA";

$taxids{$taxid}.="\t$taxid\n";

}

print $taxids{$taxid};

}

# Name: centrifuge_input_output.tsv

2 $HOME/projects/Carol_metagenomics- cacao/data/Recovery/SRA_recovered/cutadapter/cutadapter/Filtered/For0.genome_filtere d_r1.fastq $HOME/projects/Carol_metagenomics- cacao/data/Recovery/SRA_recovered/cutadapter/cutadapter/Filtered/For0.genome_filtere d_r2.fastq $HOME/projects/Carol_metagenomics- cacao/work/assembly_annotation/CENTRIFUGE/For0.out

$HOME/projects/Carol_metagenomics- cacao/work/assembly_annotation/CENTRIFUGE/For0.report.tsv

2 $HOME/projects/Carol_metagenomics- cacao/data/Recovery/SRA_recovered/cutadapter/cutadapter/Filtered/For120.genome_filte red_r1.fastq $HOME/projects/Carol_metagenomics- cacao/data/Recovery/SRA_recovered/cutadapter/cutadapter/Filtered/For120.genome_filte red_r2.fastq $HOME/projects/Carol_metagenomics- cacao/work/assembly_annotation/CENTRIFUGE/For120.out

$HOME/projects/Carol_metagenomics- cacao/work/assembly_annotation/CENTRIFUGE/For120.report.tsv

2 $HOME/projects/Carol_metagenomics- cacao/data/Recovery/SRA_recovered/cutadapter/cutadapter/Filtered/For144.genome_filte red_r1.fastq $HOME/projects/Carol_metagenomics-

cacao/data/Recovery/SRA_recovered/cutadapter/cutadapter/Filtered/For144.genome_filte red_r2.fastq $HOME/projects/Carol_metagenomics- cacao/work/assembly_annotation/CENTRIFUGE/For144.out

$HOME/projects/Carol_metagenomics- cacao/work/assembly_annotation/CENTRIFUGE/For144.report.tsv

2 $HOME/projects/Carol_metagenomics- cacao/data/Recovery/SRA_recovered/cutadapter/cutadapter/Filtered/For24.genome_filter ed_r1.fastq $HOME/projects/Carol_metagenomics- cacao/data/Recovery/SRA_recovered/cutadapter/cutadapter/Filtered/For24.genome_filter ed_r2.fastq $HOME/projects/Carol_metagenomics- cacao/work/assembly_annotation/CENTRIFUGE/For24.out

$HOME/projects/Carol_metagenomics- cacao/work/assembly_annotation/CENTRIFUGE/For24.report.tsv

2 $HOME/projects/Carol_metagenomics- cacao/data/Recovery/SRA_recovered/cutadapter/cutadapter/Filtered/For48.genome_filter ed_r1.fastq $HOME/projects/Carol_metagenomics- cacao/data/Recovery/SRA_recovered/cutadapter/cutadapter/Filtered/For48.genome_filter ed_r2.fastq $HOME/projects/Carol_metagenomics- cacao/work/assembly_annotation/CENTRIFUGE/For48.out

$HOME/projects/Carol_metagenomics- cacao/work/assembly_annotation/CENTRIFUGE/For48.report.tsv

2 $HOME/projects/Carol_metagenomics- cacao/data/Recovery/SRA_recovered/cutadapter/cutadapter/Filtered/For72.genome_filter ed_r1.fastq $HOME/projects/Carol_metagenomics- cacao/data/Recovery/SRA_recovered/cutadapter/cutadapter/Filtered/For72.genome_filter ed_r2.fastq $HOME/projects/Carol_metagenomics- cacao/work/assembly_annotation/CENTRIFUGE/For72.out

$HOME/projects/Carol_metagenomics- cacao/work/assembly_annotation/CENTRIFUGE/For72.report.tsv

2 $HOME/projects/Carol_metagenomics- cacao/data/Recovery/SRA_recovered/cutadapter/cutadapter/Filtered/For96.genome_filter ed_r1.fastq $HOME/projects/Carol_metagenomics- cacao/data/Recovery/SRA_recovered/cutadapter/cutadapter/Filtered/For96.genome_filter ed_r2.fastq $HOME/projects/Carol_metagenomics- cacao/work/assembly_annotation/CENTRIFUGE/For96.out

$HOME/projects/Carol_metagenomics- cacao/work/assembly_annotation/CENTRIFUGE/For96.report.tsv

2 $HOME/projects/Carol_metagenomics- cacao/data/Recovery/SRA_recovered/cutadapter/cutadapter/Filtered/Hib0.genome_filtere d_r1.fastq $HOME/projects/Carol_metagenomics- cacao/data/Recovery/SRA_recovered/cutadapter/cutadapter/Filtered/Hib0.genome_filtere d_r2.fastq $HOME/projects/Carol_metagenomics- cacao/work/assembly_annotation/CENTRIFUGE/Hib0.out

$HOME/projects/Carol_metagenomics- cacao/work/assembly_annotation/CENTRIFUGE/Hib0.report.tsv

2 $HOME/projects/Carol_metagenomics- cacao/data/Recovery/SRA_recovered/cutadapter/cutadapter/Filtered/Hib120.genome_filte

red_r1.fastq $HOME/projects/Carol_metagenomics- cacao/data/Recovery/SRA_recovered/cutadapter/cutadapter/Filtered/Hib120.genome_filte red_r2.fastq $HOME/projects/Carol_metagenomics- cacao/work/assembly_annotation/CENTRIFUGE/Hib120.out

$HOME/projects/Carol_metagenomics- cacao/work/assembly_annotation/CENTRIFUGE/Hib120.report.tsv

2 $HOME/projects/Carol_metagenomics- cacao/data/Recovery/SRA_recovered/cutadapter/cutadapter/Filtered/Hib144.genome_filte red_r1.fastq $HOME/projects/Carol_metagenomics- cacao/data/Recovery/SRA_recovered/cutadapter/cutadapter/Filtered/Hib144.genome_filte red_r2.fastq $HOME/projects/Carol_metagenomics- cacao/work/assembly_annotation/CENTRIFUGE/Hib144.out

$HOME/projects/Carol_metagenomics- cacao/work/assembly_annotation/CENTRIFUGE/Hib144.report.tsv

2 $HOME/projects/Carol_metagenomics- cacao/data/Recovery/SRA_recovered/cutadapter/cutadapter/Filtered/Hib24.genome_filter ed_r1.fastq $HOME/projects/Carol_metagenomics- cacao/data/Recovery/SRA_recovered/cutadapter/cutadapter/Filtered/Hib24.genome_filter ed_r2.fastq $HOME/projects/Carol_metagenomics- cacao/work/assembly_annotation/CENTRIFUGE/Hib24.out

$HOME/projects/Carol_metagenomics- cacao/work/assembly_annotation/CENTRIFUGE/Hib24.report.tsv

2 $HOME/projects/Carol_metagenomics- cacao/data/Recovery/SRA_recovered/cutadapter/cutadapter/Filtered/Hib48.genome_filter ed_r1.fastq $HOME/projects/Carol_metagenomics- cacao/data/Recovery/SRA_recovered/cutadapter/cutadapter/Filtered/Hib48.genome_filter ed_r2.fastq $HOME/projects/Carol_metagenomics- cacao/work/assembly_annotation/CENTRIFUGE/Hib48.out

$HOME/projects/Carol_metagenomics- cacao/work/assembly_annotation/CENTRIFUGE/Hib48.report.tsv

2 $HOME/projects/Carol_metagenomics- cacao/data/Recovery/SRA_recovered/cutadapter/cutadapter/Filtered/Hib72.genome_filter ed_r1.fastq $HOME/projects/Carol_metagenomics- cacao/data/Recovery/SRA_recovered/cutadapter/cutadapter/Filtered/Hib72.genome_filter ed_r2.fastq $HOME/projects/Carol_metagenomics- cacao/work/assembly_annotation/CENTRIFUGE/Hib72.out

$HOME/projects/Carol_metagenomics- cacao/work/assembly_annotation/CENTRIFUGE/Hib72.report.tsv

2 $HOME/projects/Carol_metagenomics- cacao/data/Recovery/SRA_recovered/cutadapter/cutadapter/Filtered/Hib96.genome_filter ed_r1.fastq $HOME/projects/Carol_metagenomics- cacao/data/Recovery/SRA_recovered/cutadapter/cutadapter/Filtered/Hib96.genome_filter ed_r2.fastq $HOME/projects/Carol_metagenomics- cacao/work/assembly_annotation/CENTRIFUGE/Hib96.out

$HOME/projects/Carol_metagenomics- cacao/work/assembly_annotation/CENTRIFUGE/Hib96.report.tsv

# Name: classify_scaffolds.sh

#! /bin/bash

#AUTHOR:GIOVANNI MARQUES DE CASTRO

#Used to classify the assembled sequences # merges the result of Kaiju and Centrifuge

taxdir="~/genomas_and_databases/NCBItaxonomy/"; KAIJUdir="~/genomas_and_databases/bacteria-archaea-fungi_virus/KAIJU/"; #indexes CentrifugeDir="~/genomas_and_databases/bacteria-archaea-fungi_virus/CENTRIFUGE/"; CPU=20;

for Sample in [FH]*[0-9]; do

sed "s/^>/>$Sample/" $Sample/*/scaffolds.fasta > ${Sample}-scaffolds.fasta

done

cat *-scaffolds.fasta > allsamples_scaffolds.fasta rm *-scaffolds.fasta scaf=allsamples_scaffolds.fasta out=allsamples.centrifuge

kout=allsamples.kaiju

/usr/bin/time -v centrifuge -p $CPU \

-x ${CentrifugeDir}/centrifuge-bac-arc-vir-fung \

-k 1 \

-f \

-U $scaf \

-S ${out}.out --report-file ${out}.report.tsv

/usr/bin/time -v kaiju -t ${taxdir}/nodes.dmp -f ${KAIJUdir}/genomes_sixframes.fmi \

-i $scaf -o ${kout}.tsv -z $CPU -x -E 0.01

sort --parallel=4 -k2,2 ${kout}.tsv > ${kout}.sorted.tsv

###Combine CENTRIFUGE and KAIJU outputs CENTRIFUGE_results=${out}.out; KAIJU_results=$kout;

rm ${KAIJU_results}.tsv

#Convert The regex is to correct an index bug. Should be removed in a next version tail -n+2 ${CENTRIFUGE_results}| sort --parallel=4 -k 1,1 |\

awk '{if($2~/^PTJQ01/) {$3=462795} if($2~/PZQQ01/) {$3=1291517};

if($2=="unclassified"||$6<=50 ) {$2="U";$3=0}

else {$2="C"} ; {print $2"\t"$1"\t"$3}}' > ${CENTRIFUGE_results}.kaijuformat.tsv #Merge

kaiju-mergeOutputs -v -i ${CENTRIFUGE_results}.kaijuformat.tsv -j

${KAIJU_results}.sorted.tsv -o ${KAIJU_results}.combined.tsv

#Generate summary

kaiju2table -t ${taxdir}/nodes.dmp -n ${taxdir}/names.dmp -r phylum -c 2 -p -o

$KAIJU_results.summary.txt $KAIJU_results.combined.tsv

# get last taxons, avoid ambiguity of taxa names

awk -F ";" '{if (NF-2==-1) print $0 ; else {print $1";"$2";"$(NF-2)";"$(NF-1)} } '

$KAIJU_results.summary.txt > $KAIJU_results.summary2.txt sed -i 's/^---*\n//' $KAIJU_results.summary2.txt

echo "FINISHED CONTIG CLASSIFICATION"

for i in *combined.tsv;do kaiju2krona -t ${taxdir}/nodes.dmp -n ${taxdir}/names.dmp -i $i -o

$i.kronainput -u;done

ktImportText -o META_Cacao.html *kronainput

# Name: classify_v3.sh

#! /bin/bash taxdir="/home/giovannimc/genomas_and_databases/NCBItaxonomy/"; KAIJUdir="/home/giovannimc/genomas_and_databases/bacteria-archaea- fungi_virus/100aa_KAIJU-n5/"; CentrifugeDir="/home/giovannimc/genomas_and_databases/bacteria-archaea- fungi_virus/CENTRIFUGE_08-2019/";

CPU=55;

/usr/bin/time -v centrifuge -p $CPU \

-x ${CentrifugeDir}/centrifuge-bac-arc-vir-fung \

-k 1 \

--sample-sheet centrifuge_input_output.tsv #reads and results paths

paste <(cut -f 2,3,4 centrifuge_input_output.tsv) results_kaiju.txt |\ while IFS="$(printf '\t')" read -r R1 R2 CENTRIFUGE_results KAIJU_results do

/usr/bin/time -v kaiju -t ${taxdir}/nodes.dmp -f ${KAIJUdir}/genomes_sixframes.fmi \

-i $R1 -j $R2 -o $KAIJU_results.tsv -z $CPU -a greedy -e 1 -x

sort --parallel=4 -k2,2 $KAIJU_results.tsv > $KAIJU_results.sorted.tsv # rm $KAIJU_results.tsv

###Combine CENTRIFUGE and KAIJU outputs

#Convert ##The regex is to correct an index bug. Should be removed in a next version tail -n+2 ${CENTRIFUGE_results}| sort --parallel=4 -k 1,1 |\

awk '{if($2~/^PTJQ01/) {$3=462795} if($2~/PZQQ01/) {$3=1291517};

if($2=="unclassified"||$6<=50 ) {$2="U";$3=0}

else {$2="C"} ; {print $2"\t"$1"\t"$3}}' > ${CENTRIFUGE_results}.kaijuformat.tsv

#Merge

kaiju-mergeOutputs -v -i ${CENTRIFUGE_results}.kaijuformat.tsv -j

$KAIJU_results.sorted.tsv -o $KAIJU_results.combined.tsv

#Generate summary

kaiju2table -t ${taxdir}/nodes.dmp -n ${taxdir}/names.dmp -r phylum -c 2 -p -o

$KAIJU_results.summary.txt $KAIJU_results.combined.tsv

# get last taxons, avoid ambiguity of taxa names

awk -F ";" '{if (NF-2==-1) print $0 ; else {print $1";"$2";"$(NF-2)";"$(NF-1)} } '

$KAIJU_results.summary.txt > $KAIJU_results.summary2.txt sed -i 's/^---*\n//' $KAIJU_results.summary2.txt

done

echo "FINISHED READS CLASSIFICATION"

for i in *combined.tsv;do kaiju2krona -t ${taxdir}/nodes.dmp -n ${taxdir}/names.dmp -i $i -o

$i.kronainput -u;done

ktImportText -o META_Cacao.html *kronainput

# Name: clean.sh

#!/bin/bash TruSeq_Adapter="GATCGGAAGAGCACACGTCTGAACTCCAGTCACNNNNNNNNATCTCGTATGC CGTCTTCTGCTTGAAAAAA"; #R1 NoBarcode1="GATCGGAAGAGCACACGTCTGAACTCCAGTCACATCTCGTATGCCGTCTTCTGCTTG AAAAAA";

Illumina_Single_End_PCR_Primer="GATCGGAAGAGCGTCGTGTAGGGAAAGANNNNNNNNGT GTAGATCTCGGTGGTCGCCGTATCATTAAAAAA"; #R2 NoBarcode2="GATCGGAAGAGCGTCGTGTAGGGAAAGAGTGTAGATCTCGGTGGTCGCCGTATCA TTAAAAAA";

Illumina_universal_primer="AATGATACGGCGACCACCGAGATCTACACTCTTTCCCTACACGACG CTCTTCCGATCT";

#for short reference IUP=$Illumina_universal_primer;

TSA=$TruSeq_Adapter; IPCR=$Illumina_Single_End_PCR_Primer;

host_DB="~/genomas_and_databases/cacau/bowtie2index/Theobroma_cacao"; mkdir cutadapter ;

mkdir cutadapter/Filtered

mkdir cutadapter/Filtered/Cleaned; for r1 in raw/*1.fq.gz

do

r2=${r1%1.fq.gz}2.fq.gz; out=${r1:4};

out=${out%_1.fq.gz}; echo $out;

cutadapt --interleaved -j 10 -a $NoBarcode1 -A $NoBarcode2 -g $TSA -G $IPCR $r1

$r2 |\

cutadapt --interleaved -a A${TSA} -A A${IPCR} -a $IUP -A $IUP -j 8 \

-o cutadapter/${out}_NOadapter_1.fastq -p cutadapter/${out}_NOadapter_2.fastq -

out1=$out.genome_filtered_r1.fastq; out2=$out.genome_filtered_r2.fastq;

bowtie2 -p 20 -x $host_DB -1 cutadapter/${out}_NOadapter_1.fastq -2 cutadapter/${out}_NOadapter_2.fastq | samtools view -bS - -o cutadapter/Filtered/$out.bam

samtools view -F 12 cutadapter/Filtered/$out.bam -o cutadapter/Filtered/$out.mapped.12.bam

samtools view -F 4 -f 8 cutadapter/Filtered/$out.bam -o cutadapter/Filtered/$out.mapped.4-8.bam

samtools view -F 8 -f 4 cutadapter/Filtered/$out.bam -o cutadapter/Filtered/$out.mapped.8-4.bam

samtools merge cutadapter/Filtered/$out.mapped.bam cutadapter/Filtered/$out.mapped.*.bam

rm cutadapter/Filtered/$out.mapped.*.bam

samtools view -f 12 -F 256 cutadapter/Filtered/$out.bam | samtools sort --threads 6

-n - -o cutadapter/Filtered/$out.bothENDs_unmapped_sorted.bam

bedtools bamtofastq -i cutadapter/Filtered/$out.bothENDs_unmapped_sorted.bam

-fq cutadapter/Filtered/$out1 -fq2 cutadapter/Filtered/$out2 # rm $out.bam

flash -x 0.03 -M 50 -o cutadapter/Filtered/${out}.noadapt.filtered -t 8 cutadapter/Filtered/$out1 cutadapter/Filtered/$out2

done

#cd cutadapter/Filtered/Cleaned #mkdir FASTQC-NoAdapter

#fastqc -t 5 -o FASTQC-NoAdapter *fastq

# Name: get_bins-and-assembly.sh

#! /bin/bash

#AUTHOR:GIOVANNI MARQUES DE CASTRO

source ~/.bashrc conda deactivate #conda activate CPU=10

datadir="~/projects/Carol_metagenomics- cacao/data/Recovery/SRA_recovered/cutadapter/cutadapter/Filtered" # Dir with the Reads after cutting the adapter and host filtering KAIJU_result_dir="~/projects/Carol_metagenomics- cacao/work/assembly_annotation/KAIJU"; # Dir with the results combined from Centrifuge and Kaiju (*combined.tsv)

for R1 in ${datadir}/H*.noadapt.filtered.notCombined_1.fastq do

######### SETTING VARIABLES

R2=${R1%_1.fastq}_2.fastq merged=${R1%notCombined_1.fastq}extendedFrags.fastq

SAMPLE=`echo "${R1%.noadapt.filtered.notCombined_1.fastq}"| awk -F '/' '{print $NF}'` r1_trimmed="${R1%.fastq}.trim.fastq"

r2_trimmed="${R2%.fastq}.trim.fastq" r1_trimmed_unpaired="${R1%.fastq}.trim_unpaired.fastq" r2_trimmed_unpaired="${R2%.fastq}.trim_unpaired.fastq" r1r2_single_trimmed="${R1%_1.fastq}.trim_unpaired.fastq" merged_trimed="${merged%.fastq}.trim.fastq"

mkdir $SAMPLE cd $SAMPLE

########### TRIMMING

java -jar ~/bin/Trimmomatic-0.38/trimmomatic-0.38.jar PE -threads $CPU -phred33 \

$R1 \

$R2 \

$r1_trimmed \

$r1_trimmed_unpaired \

$r2_trimmed \

$r2_trimmed_unpaired \

LEADING:15 TRAILING:15 SLIDINGWINDOW:5:15 MINLEN:50

java -jar ~/bin/Trimmomatic-0.38/trimmomatic-0.38.jar SE -threads $CPU -phred33 \

$merged \

$merged_trimed \

LEADING:15 TRAILING:15 SLIDINGWINDOW:5:15 MINLEN:50

cat $r1_trimmed_unpaired $r2_trimmed_unpaired > $r1r2_single_trimmed

########## REMOVING some tmp files

rm $r1_trimmed_unpaired $r2_trimmed_unpaired ######### ASSEMBLING

#bin by phylum

/usr/bin/time -v perl ~/projects/Carol_metagenomics-cacao/bin/get_bins.pl -c

$KAIJU_result_dir/${SAMPLE}.out.combined.tsv -r1 $r1_trimmed -r2 $r2_trimmed -merged

$merged_trimed -s $r1r2_single_trimmed for R1_BINNED in *.R1.fastq

do pwd

R2_BINNED=${R1_BINNED%R1.fastq}R2.fastq SINGLE_BINNED=${R1_BINNED%R1.fastq}single.fastq MERGED_BINNED=${R1_BINNED%R1.fastq}merged.fastq ASSEMBLY_OUT=${R1_BINNED%.R1.fastq}

spades.py -m 300 -t $CPU --meta -1 $R1_BINNED -2 $R2_BINNED --merged

$MERGED_BINNED -s $SINGLE_BINNED -o $ASSEMBLY_OUT

done cd .. done

# Name: get_bins.pl

#! /bin/env perl

#AUTHOR:GIOVANNI MARQUES DE CASTRO

#TESTED on illumina paired end reads (hiseq)

use local::lib; use Bio::Taxon;

use Bio::Tree::Tree; use Getopt::Long; use warnings;

use strict;

my ($classified_reads,$read1,$read2,$single,$merged) = ("")x5; my $rank='phylum';

my $usage="USAGE:\nperl $0 -c CLASSIFICATION_FILE(KAIJU_LIKE) -r1 READ_1.fastq -r2 READ_2.fastq -s Single.fastq -merged Extendend.fastq [optional: -rank phylum]\n"; #my $tree_functions = Bio::Tree::Tree->new();

GetOptions (#"length=i" => \$length, # numeric "classification_file=s" => \$classified_reads, # string "r1=s" => \$read1, # string

"r2=s" => \$read2, # string "single=s"=>\$single, "merged=s"=>\$merged,

"rank=s"=>\$rank #string (defaults to phylum) # "verbose" => \$verbose # flag

)||die "$usage";

open(CLASSIFIED_READS,"<$classified_reads")||die "can not open '$classified_reads'\n$usage";

if ($read1 ne '') { open(READ1,"<$read1") || die "cannot open read1 '$read1'\n$usage" };

if ($read2 ne '') { open(READ2,"<$read2") || die "cannot open read2 '$read2'\n$usage" }; #BUG: it runs if named pipe is empty

if ($single ne ''){ open(SINGLE,"<$single")|| die "cannot open single '$single'\n$usage"} ; if ($merged ne ''){ open(MERGED,"<$merged")|| die "cannot open merged '$merged'\n$usage"} ;

if (($read1 eq '' || $read2 eq '') && $merged eq '' && $single eq '') {die "An input is missing! A pair or a single is needed!\n$usage"};

# Get one from a database

my $dbh = Bio::DB::Taxonomy->new(-source => 'flatfile',

-directory=> '/home/giovannimc/genomas_and_databases/NCBItaxonomy/BioTaxon',

-nodesfile=> '/home/giovannimc/genomas_and_databases/NCBItaxonomy/nodes.dmp',

-namesfile=> '/home/giovannimc/genomas_and_databases/NCBItaxonomy/names.dmp'); my $taxon = $dbh->get_taxon(-taxonid => '9606'); #test if the db is working my %hashing;#accelerate the search

my %taxa_read;

my %filehandles; # to write multiple files while(<CLASSIFIED_READS>){

if( $_=~/^C\t(.*)\t(.*)$/){ my $read=$1;

my $taxid=$2;

if($hashing{$taxid}){ #has seen the taxid, avoid bio:tree fucntions

$taxa_read{$read}=$hashing{$taxid};

}else{

$taxon = $dbh->get_taxon(-taxonid => $taxid);

my $tree = Bio::Tree::Tree->new(-node => $taxon);

my $superkingdom =$tree->find_node(-rank => 'superkingdom');#to

have remember better

my $node = $tree->find_node(-rank => $rank); if($node){ #node has phylum, or any other desired rank

my $TAXON=$node->scientific_name;

$TAXON=~s/ /_/g; #some taxons have spaces

$superkingdom = $superkingdom->scientific_name;

$hashing{$taxid}=$TAXON; #hashing to use Bio::Taxon and BIO::Tree::Tree once per desired taxon

$taxa_read{$read}=$TAXON; if(!$filehandles{"$TAXON.R1"}){

open($filehandles{"$TAXON.R1"},">$superkingdom.$TAXON.R1.fastq")||die "CANNOT open $TAXON.R1.fastq to write\n";

open($filehandles{"$TAXON.R2"},">$superkingdom.$TAXON.R2.fastq")||die "CANNOT open $TAXON.R2.fastq to write\n";

open($filehandles{"$TAXON.s"},">$superkingdom.$TAXON.single.fastq")||die "CANNOT open $TAXON.single.fastq to write\n";

open($filehandles{"$TAXON.m"},">$superkingdom.$TAXON.merged.fastq")||die "CANNOT open $TAXON.merged.fastq to write\n";

}

}

}

}

}

while(<READ1>){

my$r1=$_;

$r1=~s/^@|\/1$//g; chomp($r1); if($taxa_read{$r1}){

print {$filehandles{"$taxa_read{$r1}.R1"}} ( $_

.<READ1>.<READ1>.<READ1>);#dereference the filehandle to print print {$filehandles{"$taxa_read{$r1}.R2"}}

(<READ2>.<READ2>.<READ2>.<READ2>);

}else{ #read pair not classified

<READ1>.<READ1>.<READ1>;#jump 3 lines

<READ2>.<READ2>.<READ2>.<READ2>;#jump 4 lines

}

}

if ($single ne ''){

while(<SINGLE>){

my$r1=$_;

$r1=~s/^@|\/1$//g; chomp($r1); if($taxa_read{$r1}){

print {$filehandles{"$taxa_read{$r1}.s"}} ( $_

.<SINGLE>.<SINGLE>.<SINGLE>);#dereference the filehandle to print

}

else{

<SINGLE>.<SINGLE>.<SINGLE>;#jump 3 lines

}

}

}

if ($merged ne ''){

while(<MERGED>){

my$r1=$_;

$r1=~s/^@|\/1$//g; chomp($r1); if($taxa_read{$r1}){

print {$filehandles{"$taxa_read{$r1}.m"}} ( $_

.<MERGED>.<MERGED>.<MERGED>);#dereference the filehandle to print

}

else{

<MERGED>.<MERGED>.<MERGED>;#jump 3 lines

}

}

}

# Name: Interpro_annot_and_join_quant.sh

#! /bin/bash

#AUTHOR:GIOVANNI MARQUES DE CASTRO

threads=30

#This generates the commands to use as input for the qsub, so that qsub or parallel for SAMPLE in For*/ Hib*/

do

cd $SAMPLE

for prok_PHYLUM in Bacteria.*/ Archaea*/ do

goterms;"

cd $prok_PHYLUM

if [ -e scaffolds.fasta ] then

CW=`pwd`

echo "cd $CW ;\

~/bin/prodigal -p meta -i scaffolds.fasta -o Prodigal -a Prodigal.aa;\ sed -i 's/\*//' Prodigal.aa;\

interproscan.sh -i Prodigal.aa --cpu $threads -b Interproscan -dp -pa -

done

fi cd ..

for euk_phylum in Eukaryota*/ do

cd $euk_phylum if [ -e scaffolds.fasta ] then

CW=`pwd` echo "cd $CW;\

augustus --species=generic scaffolds.fasta > augustus_generic.gff;\ getAnnoFasta.pl augustus_generic.gff;\

-dp -pa -goterms;"

fi cd ..

interproscan.sh -i augustus_generic.aa --cpu $threads -b Interproscan

done

cd ..

done > annotation_commands.sh

sh annotation_commands.sh #parallel works here #Get all lines with EC numbers

#join KEGGS and GOs with same id #get contig name from gene prediction

grep "[^:][0-9]\.[0-9]\+\.[0-9]\+\.[0-9]\+" */*/Interproscan.tsv > EC_KEGG_interpro.tsv cut -f 1,14,15 EC_KEGG_interpro.tsv | uniq | perl join_go_keeg.pl - | perl add_contig_name_and_quant.pl - > EC_KEGG_interpro.cds2node.tsv

#get abundance of each contig

head -n1 For0_quant/quant.sf |cut -f 1,4,5 | sed 's/Name\t/Sample\tID\t/' >

EC_KEGG_quant.tsv for i in *quant/

do

sample=${i%_quant/};

grep -wFf <(grep -w "^$sample" EC_KEGG_interpro.cds2node.tsv|cut -f 4)

$i/quant.sf | cut -f 1,4,5| sed "s|^|$sample\t|" >> EC_KEGG_quant.tsv done

# Name: join_go_keeg.pl

#! /bin/env perl

#AUTHOR:GIOVANNI MARQUES DE CASTRO

#Get the tsv from interpro and place the GO and KEGG anotations in two columns use warnings;

use strict;

open(IN,$ARGV[0])||die "cannot open file $ARGV[0]\n" ; #EC_KEGG_interpro.tsv my $c=0;

my @mem=split(/\t/,<IN>); chomp(@mem); while(<IN>){

my @line=split(/\t/,$_); chomp(@line); if($line[0] eq $mem[0]){

$c=1;

my %uniq_go; my %uniq_kegg;

my @GO =split(/\|/,$line[1]); my @KEGG=split(/\|/,$line[2]);

foreach my $go ( @GO ) {

$uniq_go{$go} ='';

}

foreach my $kegg ( @KEGG ) {

$uniq_kegg{$kegg}='';

}

@GO =split(/\|/,$mem[1]); @KEGG=split(/\|/,$mem[2]); foreach my $go (@GO){

$uniq_go{$go} ='';

}

foreach my $kegg (@KEGG){

$uniq_kegg{$kegg}='';

}

my $go=join "|", keys %uniq_go;

my $kegg=join "|", keys %uniq_kegg; # $line[0]."\t$go\t$kegg\n"; @mem=($line[0],$go,$kegg);

if(eof){

}

else{

}

}

if(eof){

}else{

}

$c=0

print join("\t",@mem)."\n";

print join("\t",@mem)."\n"; print $_;

print join("\t",@mem)."\n";

@mem=@line;

}

# Name: quant2.sh

#!/ bin/bash datadir="/home/giovannimc/projects/Carol_metagenomics- cacao/data/Recovery/SRA_recovered/cutadapter/cutadapter/Filtered";

#First, join all scaffolds in one file to index rm allscaffolds.fasta

for i in Hib0/ Hib24/ Hib48/ Hib72/ Hib96/ Hib120/ Hib144/ For120/ For144/ For0/ For24/ For48/ For72/ For96/;do sed "s/>/>$i" ${i}*/scaffolds.fasta >> allscaffolds.fasta;done

salmon index -t allscaffolds.fasta -p 15 -i allscaffolds_salmon.index

#now, quantify

for i in Hib0/ Hib24/ Hib48/ Hib72/ Hib96/ Hib120/ Hib144/ For120/ For144/ For0/ For24/ For48/ For72/ For96/

do

salmon quant -p 20 --numBootstraps 100 -l IU --meta -o ${i%/}_quant -1

${datadir}/${i%/}.genome_filtered_r1.fastq.gz -2

${datadir}/${i%/}.genome_filtered_r2.fastq.gz -i allscaffolds_salmon.index done

head -n 1 For0_quant/quant.sf > EC_KEGG_quant.tsv

grep -Ff <(cut -f 1 EC_KEGG_interpro.tsv| sed 's/.*\(NODE.*\)_[0-9]\+/\1/')

*_quant/quant.sf | cut -f 1,3,4 >> EC_KEGG_quant.tsv

# Name: Run_all_Assembly_annotate_quantify.sh

#! /bin/bash

#AUTHOR:GIOVANNI MARQUES DE CASTRO

#assumes cleaned reads are in the right dir classify_v3.sh #centrifuge and Kaiju

get_bins-and-assembly.sh #call get_bin.pl, Trimmomatic, Spades quant2.sh #call salmon

Interpro_annot_and_join_quant.sh #calls Prodigal, Augustus, Interproscan, join_go_keeg.pl

, add_contig_name_and_quant.pl classify_scaffolds.sh #call Centrifuge and Kaiju,
