# Supplementary material for: Unraveling potential enzymes and their functional role in fine cocoa beans fermentation using temporal shotgun metagenomics
Source: Front Microbiol. 2022 Nov 3;13:994524. doi: 10.3389/fmicb.2022.994524 (PMC9671152; doi:10.3389/fmicb.2022.994524)
Supplement: Supplementary file 2 [file Table_2.DOCX]

dados_ECs.R

| ## | Individuals | (the 10 first) |  | | | | | |
| --- | --- | --- | --- | --- | --- | --- | --- | --- |
| ## |  | Dist Dim.1 | ctr | cos2 |  | Dim.2 | ctr | cos2 |
| ## | For0 | \| 41.442 \| -34.327 | 16.157 | 0.686 | \| | -7.958 | 4.001 | 0.037 |
| ## | For24 | \| 25.450 \| -16.809 | 3.874 | 0.436 | \| | -6.467 | 2.642 | 0.065 |
| ## | For48 | \| 21.533 \| -12.391 | 2.105 | 0.331 | \| | -8.568 | 4.638 | 0.158 |
| ## | For72 | \| 24.558 \| 12.489 | 2.139 | 0.259 | \| | -10.217 | 6.595 | 0.173 |
| ## | For96 | \| 27.522 \| 8.165 | 0.914 | 0.088 | \| | -16.403 | 16.997 | 0.355 |
| ## | For120 | \| 31.481 \| 27.109 | 10.077 | 0.742 | \| | 4.179 | 1.103 | 0.018 |
| ## | For144 | \| 41.651 \| 29.874 | 12.238 | 0.514 | \| | 20.534 | 26.636 | 0.243 |

*ricardosolar*

**setwd**("~/Dropbox/UFMG/Artigos/Ari_Cacau/")

dados <- **read.table**("dados_ECs.txt", h=T, row.names = 1) dados2 <- **t**(dados)

**library**(vegan) **library**(ggplot2) **library**(factoextra) **library**(ade4) **library**(FactoMineR)

##First working PCAs

res.PCA <- FactoMineR**::PCA**(dados2, scale.unit = T, graph = F, ncp=**nrow**(dados2))

##scaling is TRUE, for PCAS abundances are advised to be scaled to zero mean and unit variance

**summary**(res.PCA)

##

## Call:

## FactoMineR::PCA(X = dados2, scale.unit = T, ncp = nrow(dados2), ## graph = F)

## ##

## Eigenvalues

## Dim.1 Dim.2 Dim.3 Dim.4 Dim.5 Dim.6

| ## | Variance |  |  |  | 520.916 | 113.068 | 71.792 | 55.646 | 35.515 | 30.692 |
| --- | --- | --- | --- | --- | --- | --- | --- | --- | --- | --- |
| ## | % of var. |  |  |  | 55.240 | 11.990 | 7.613 | 5.901 | 3.766 | 3.255 |
| ## | Cumulative | % | of | var. | 55.240 | 67.231 | 74.844 | 80.745 | 84.511 | 87.766 |
| ## |  |  |  |  | Dim.7 | Dim.8 | Dim.9 | Dim.10 | Dim.11 | Dim.12 |
| ## | Variance |  |  |  | 25.686 | 22.194 | 19.125 | 15.059 | 12.359 | 11.684 |
| ## | % of var. |  |  |  | 2.724 | 2.354 | 2.028 | 1.597 | 1.311 | 1.239 |
| ## | Cumulative | % | of | var. | 90.489 | 92.843 | 94.871 | 96.468 | 97.779 | 99.018 |
| ## |  |  |  |  | Dim.13 |  | | | | |
| ## | Variance |  |  |  | 9.262 |  |  |  |  |  |
| ## | % of var. |  |  |  | 0.982 |  |  |  |  |  |
| ## | Cumulative | % | of | var. | 100.000 |  |  |  |  |  |
| ## |  |  |  |  |  |  |  |  |  |  |

| ## | Hib0 | \| | 44.611 | \| -38.573 | 20.402 | | 0.748 \| | 14.513 | 13.306 | 0.106 |
| --- | --- | --- | --- | --- | --- | --- | --- | --- | --- | --- |
| ## | Hib24 | \| | 34.171 | \| -23.077 | 7.302 | | 0.456 \| | 14.787 | 13.812 | 0.187 |
| ## | Hib48 | \| | 23.964 | \| -19.690 | 5.316 | | 0.675 \| | 4.037 | 1.030 | 0.028 |
| ## |  |  | Dim.3 | ctr | cos2 | |  |  |  |  |
| ## | For0 | \| | -17.053 | 28.934 | 0.169 | \| | | | | |
| ## | For24 | \| | -5.064 | 2.551 | 0.040 | \| | | | | |
| ## | For48 | \| | -6.888 | 4.720 | 0.102 | \| | | | | |
| ## | For72 | \| | 7.920 | 6.241 | 0.104 | \| | | | | |
| ## | For96 | \| | 8.227 | 6.734 | 0.089 | \| | | | | |
| ## | For120 | \| | -3.918 | 1.528 | 0.015 | \| | | | | |
| ## | For144 | \| | -14.133 | 19.874 | 0.115 | \| | | | | |
| ## | Hib0 | \| | 5.414 | 2.917 | 0.015 | \| | | | | |
| ## | Hib24 | \| | 15.020 | 22.446 | 0.193 | \| | | | | |
| ## | Hib48 | \| | 1.556 | 0.241 | 0.004 | \| | | | | |
| ## |  |  |  |  |  |  | | | | |
| ## Variables (the 10 first) | | | | | | | | | | |

| ## Dim.1 ctr cos2 Dim.2 ctr cos2 ## 1.1.1.1 \| 0.943 0.171 0.889 \| 0.209 0.039 0.044 | | | | | | | | | | \| | Dim.3 0.146 | ctr 0.029 |
| --- | --- | --- | --- | --- | --- | --- | --- | --- | --- | --- | --- | --- |
| ## | 1.1.1.100 | \| | 0.925 | 0.164 | 0.856 | \| | 0.086 | 0.007 | 0.007 | \| | 0.230 | 0.074 |
| ## | 1.1.1.103 | \| | -0.882 | 0.149 | 0.778 | \| | 0.377 | 0.126 | 0.142 | \| | 0.180 | 0.045 |
| ## | 1.1.1.127 | \| | -0.726 | 0.101 | 0.527 | \| | 0.137 | 0.017 | 0.019 | \| | 0.308 | 0.132 |
| ## | 1.1.1.130 | \| | -0.547 | 0.057 | 0.299 | \| | 0.506 | 0.226 | 0.256 | \| | 0.476 | 0.315 |
| ## | 1.1.1.169 | \| | 0.784 | 0.118 | 0.614 | \| | 0.568 | 0.286 | 0.323 | \| | 0.019 | 0.001 |
| ## | 1.1.1.17 | \| | 0.821 | 0.129 | 0.674 | \| | 0.367 | 0.119 | 0.135 | \| | 0.400 | 0.223 |
| ## | 1.1.1.18 | \| | -0.869 | 0.145 | 0.755 | \| | 0.448 | 0.178 | 0.201 | \| | 0.123 | 0.021 |
| ## | 1.1.1.193 | \| | 0.924 | 0.164 | 0.854 | \| | 0.241 | 0.052 | 0.058 | \| | 0.103 | 0.015 |
| ## | 1.1.1.205 | \| | 0.771 | 0.114 | 0.594 | \| | 0.261 | 0.060 | 0.068 | \| | 0.106 | 0.016 |
| ## |  |  | cos2 |  |  |  |  |  |  |  |  |  |

| ## | 1.1.1.1 | 0.021 | \| |
| --- | --- | --- | --- |
| ## | 1.1.1.100 | 0.053 | \| |
| ## | 1.1.1.103 | 0.033 | \| |
| ## | 1.1.1.127 | 0.095 | \| |
| ## | 1.1.1.130 | 0.226 | \| |
| ## | 1.1.1.169 | 0.000 | \| |
| ## | 1.1.1.17 | 0.160 | \| |
| ## | 1.1.1.18 | 0.015 | \| |
| ## | 1.1.1.193 | 0.011 | \| |
| ## | 1.1.1.205 | 0.011 | \| |
| ##Correlation between variables and all axes of PCA  **dimdesc**(res.PCA, 1**:**13, proba = 0.01) | | | |

## $Dim.1

## $Dim.1$quanti

## correlation p.value

| ## | 1.4.3.19 | 0.9863575 | 9.039070e-11 |
| --- | --- | --- | --- |
| ## | 3.5.1.108 | 0.9850327 | 1.571746e-10 |
| ## | 2.5.1.17 | 0.9837283 | 2.587644e-10 |
| ## | 5.4.2.12 | 0.9830167 | 3.340097e-10 |
| ## | 3.5.2.3 | 0.9809213 | 6.682965e-10 |
| ## | 2.3.1.31 | 0.9765573 | 2.278362e-09 |
| ## | 4.1.1.5 | 0.9747535 | 3.540667e-09 |
| ## | 3.5.4.19 | 0.9735744 | 4.644346e-09 |
| ## | 5.4.99.17 | 0.9725616 | 5.807264e-09 |
| ## | 4.2.1.129 | 0.9725616 | 5.807264e-09 |

| ## | 6.6.1.1 | 0.9720957 | 6.418057e-09 |
| --- | --- | --- | --- |
| ## | 4.2.1.126 | 0.9705460 | 8.846871e-09 |
| ## | 2.3.1.274 | 0.9686795 | 1.273900e-08 |
| ## | 2.7.4.1 | 0.9662036 | 2.000104e-08 |
| ## | 1.1.1.85 | 0.9659194 | 2.101863e-08 |
| ## | 2.4.2.17 | 0.9657686 | 2.157583e-08 |
| ## | 1.2.1.88 | 0.9639960 | 2.909724e-08 |
| ## | 4.1.99.17 | 0.9635202 | 3.144961e-08 |
| ## | 5.1.3.2 | 0.9632563 | 3.282062e-08 |
| ## | 2.7.7.70 | 0.9629873 | 3.426907e-08 |
| ## | 2.7.1.167 | 0.9629873 | 3.426907e-08 |
| ## | 2.3.1.117 | 0.9629261 | 3.460551e-08 |
| ## | 5.4.99.18 | 0.9628141 | 3.522885e-08 |
| ## | 2.1.1.195 | 0.9625565 | 3.669828e-08 |
| ## | 2.7.8.5 | 0.9624881 | 3.709666e-08 |
| ## | 4.2.1.109 | 0.9620053 | 4.001338e-08 |
| ## | 6.3.3.1 | 0.9619209 | 4.054213e-08 |
| ## | 2.1.1.13 | 0.9612362 | 4.505021e-08 |
| ## | 1.2.1.41 | 0.9593829 | 5.938012e-08 |
| ## | 2.8.1.10 | 0.9591115 | 6.176473e-08 |
| ## | 6.2.1.1 | 0.9585278 | 6.716150e-08 |
| ## | 2.7.2.8 | 0.9582712 | 6.965540e-08 |
| ## | 2.1.3.2 | 0.9580515 | 7.185026e-08 |
| ## | 2.7.4.22 | 0.9572369 | 8.049581e-08 |
| ## | 6.3.2.4 | 0.9570904 | 8.213856e-08 |
| ## | 3.1.2.6 | 0.9562881 | 9.163436e-08 |
| ## | 1.3.1.98 | 0.9558768 | 9.684358e-08 |
| ## | 2.1.1.64 | 0.9551197 | 1.070748e-07 |
| ## | 2.1.1.222 | 0.9551197 | 1.070748e-07 |
| ## | 2.10.1.1 | 0.9539667 | 1.243634e-07 |
| ## | 1.13.11.79 | 0.9538863 | 1.256500e-07 |
| ## | 2.7.6.1 | 0.9533010 | 1.353559e-07 |
| ## | 2.8.1.12 | 0.9528910 | 1.425160e-07 |
| ## | 3.6.1.31 | 0.9527146 | 1.456918e-07 |
| ## | 2.4.2.21 | 0.9516603 | 1.659185e-07 |
| ## | 2.4.99.12 | 0.9510407 | 1.788533e-07 |
| ## | 2.4.2.10 | 0.9503859 | 1.934172e-07 |
| ## | 6.1.1.21 | 0.9503112 | 1.951403e-07 |
| ## | 3.1.3.77 | 0.9501860 | 1.980538e-07 |
| ## | 1.13.11.54 | 0.9498564 | 2.058986e-07 |
| ## | 2.4.2.18 | 0.9495249 | 2.140480e-07 |
| ## | 2.3.1.275 | 0.9489849 | 2.278919e-07 |
| ## | 2.2.1.7 | 0.9483879 | 2.440488e-07 |
| ## | 2.7.1.148 | 0.9478218 | 2.602352e-07 |
| ## | 1.4.9.1 | 0.9473229 | 2.752250e-07 |
| ## | 4.2.3.5 | 0.9469346 | 2.873802e-07 |
| ## | 2.6.1.9 | 0.9468467 | 2.901925e-07 |
| ## | 2.5.1.15 | 0.9465235 | 3.007275e-07 |
| ## | 2.4.1.83 | 0.9465167 | 3.009540e-07 |
| ## | 4.1.99.22 | 0.9464605 | 3.028187e-07 |
| ## | 1.17.7.4 | 0.9459168 | 3.213599e-07 |
| ## | 2.7.4.8 | 0.9456460 | 3.309406e-07 |
| ## | 2.7.8.26 | 0.9450216 | 3.539327e-07 |
| ## | 4.1.1.65 | 0.9441894 | 3.866120e-07 |

| ## | 4.3.2.1 | 0.9441683 | 3.874723e-07 |
| --- | --- | --- | --- |
| ## | 4.2.1.75 | 0.9441526 | 3.881149e-07 |
| ## | 2.7.7.38 | 0.9439931 | 3.946731e-07 |
| ## | 1.1.1.1 | 0.9429306 | 4.407574e-07 |
| ## | 6.1.1.16 | 0.9428211 | 4.457482e-07 |
| ## | 2.7.1.39 | 0.9425783 | 4.569833e-07 |
| ## | 2.3.1.51 | 0.9415839 | 5.054649e-07 |
| ## | 2.8.1.6 | 0.9410899 | 5.310856e-07 |
| ## | 3.4.11.1 | 0.9410846 | 5.313645e-07 |
| ## | 2.7.1.23 | 0.9409567 | 5.381750e-07 |
| ## | 3.5.1.18 | 0.9409415 | 5.389857e-07 |
| ## | 4.2.1.51 | 0.9408433 | 5.442724e-07 |
| ## | 5.1.3.20 | 0.9407129 | 5.513488e-07 |
| ## | 4.1.1.20 | 0.9406342 | 5.556623e-07 |
| ## | 6.3.2.17 | 0.9396848 | 6.098814e-07 |
| ## | 2.5.1.47 | 0.9391692 | 6.411156e-07 |
| ## | 2.7.7.62 | 0.9386521 | 6.737495e-07 |
| ## | 2.7.1.156 | 0.9386521 | 6.737495e-07 |
| ## | 5.3.1.24 | 0.9373675 | 7.608108e-07 |
| ## | 1.3.3.3 | 0.9372635 | 7.682411e-07 |
| ## | 6.1.1.1 | 0.9371159 | 7.788991e-07 |
| ## | 2.4.1.80 | 0.9357849 | 8.806184e-07 |
| ## | 4.2.1.46 | 0.9355459 | 8.999974e-07 |
| ## | 2.7.7.24 | 0.9345196 | 9.872467e-07 |
| ## | 4.1.1.81 | 0.9344830 | 9.904836e-07 |
| ## | 2.7.8.13 | 0.9341930 | 1.016438e-06 |
| ## | 2.5.1.39 | 0.9336000 | 1.071241e-06 |
| ## | 1.5.5.2 | 0.9332974 | 1.100136e-06 |
| ## | 3.6.1.66 | 0.9319316 | 1.238676e-06 |
| ## | 6.1.1.11 | 0.9318515 | 1.247226e-06 |
| ## | 1.3.1.9 | 0.9310701 | 1.333239e-06 |
| ## | 4.99.1.1 | 0.9307553 | 1.369239e-06 |
| ## | 6.3.2.1 | 0.9298165 | 1.481394e-06 |
| ## | 2.7.4.16 | 0.9290898 | 1.573320e-06 |
| ## | 4.1.1.50 | 0.9285482 | 1.644858e-06 |
| ## | 4.3.99.3 | 0.9281831 | 1.694566e-06 |
| ## | 2.3.1.35 | 0.9279610 | 1.725402e-06 |
| ## | 2.4.1.288 | 0.9276511 | 1.769219e-06 |
| ## | 2.7.4.9 | 0.9273505 | 1.812577e-06 |
| ## | 2.4.1.227 | 0.9270798 | 1.852374e-06 |
| ## | 3.1.3.18 | 0.9269836 | 1.866696e-06 |
| ## | 2.4.2.7 | 0.9267736 | 1.898258e-06 |
| ## | 1.1.1.100 | 0.9250796 | 2.169328e-06 |
| ## | 1.3.5.2 | 0.9250098 | 2.181148e-06 |
| ## | 4.1.1.12 | 0.9248706 | 2.204874e-06 |
| ## | 2.5.1.3 | 0.9248548 | 2.207580e-06 |
| ## | 3.5.4.26 | 0.9238557 | 2.384388e-06 |
| ## | 1.1.1.193 | 0.9238557 | 2.384388e-06 |
| ## | 2.7.7.18 | 0.9236305 | 2.425798e-06 |
| ## | 6.1.1.3 | 0.9230171 | 2.541638e-06 |
| ## | 2.8.1.7 | 0.9227502 | 2.593425e-06 |
| ## | 3.5.4.16 | 0.9223070 | 2.681372e-06 |
| ## | 5.1.3.1 | 0.9221570 | 2.711688e-06 |
| ## | 6.1.1.4 | 0.9220557 | 2.732303e-06 |

| ## | 2.5.1.75 | 0.9218015 | 2.784657e-06 |
| --- | --- | --- | --- |
| ## | 3.1.3.25 | 0.9213125 | 2.887651e-06 |
| ## | 2.7.7.41 | 0.9211321 | 2.926445e-06 |
| ## | 2.6.1.42 | 0.9211121 | 2.930760e-06 |
| ## | 2.7.2.11 | 0.9209867 | 2.958004e-06 |
| ## | 5.3.1.16 | 0.9209861 | 2.958140e-06 |
| ## | 4.3.3.7 | 0.9202354 | 3.125632e-06 |
| ## | 5.1.1.7 | 0.9196859 | 3.253102e-06 |
| ## | 2.7.1.24 | 0.9196330 | 3.265611e-06 |
| ## | 6.1.1.17 | 0.9190576 | 3.404094e-06 |
| ## | 2.7.4.3 | 0.9187295 | 3.485186e-06 |
| ## | 3.6.1.9 | 0.9181769 | 3.625382e-06 |
| ## | 2.7.4.25 | 0.9180519 | 3.657721e-06 |
| ## | 2.3.1.180 | 0.9169352 | 3.957353e-06 |
| ## | 4.3.1.19 | 0.9160282 | 4.215340e-06 |
| ## | 2.4.2.14 | 0.9159650 | 4.233806e-06 |
| ## | 5.4.2.10 | 0.9156426 | 4.329118e-06 |
| ## | 1.18.1.2 | 0.9154230 | 4.395043e-06 |
| ## | 3.5.4.10 | 0.9147085 | 4.615257e-06 |
| ## | 2.1.2.3 | 0.9145609 | 4.661864e-06 |
| ## | 6.1.1.7 | 0.9140000 | 4.842515e-06 |
| ## | 2.7.1.130 | 0.9138856 | 4.880031e-06 |
| ## | 2.4.1.129 | 0.9127861 | 5.253217e-06 |
| ## | 6.3.2.5 | 0.9123996 | 5.389815e-06 |
| ## | 6.1.1.19 | 0.9122264 | 5.451970e-06 |
| ## | 4.3.2.2 | 0.9120567 | 5.513434e-06 |
| ## | 2.5.1.19 | 0.9118760 | 5.579539e-06 |
| ## | 4.1.1.97 | 0.9117401 | 5.629671e-06 |
| ## | 2.3.1.39 | 0.9115824 | 5.688279e-06 |
| ## | 7.1.1.8 | 0.9108138 | 5.981287e-06 |
| ## | 1.1.1.94 | 0.9106965 | 6.027085e-06 |
| ## | 2.1.1.148 | 0.9106410 | 6.048821e-06 |
| ## | 2.4.1.182 | 0.9105127 | 6.099401e-06 |
| ## | 6.1.1.10 | 0.9102863 | 6.189465e-06 |
| ## | 6.3.2.6 | 0.9096967 | 6.429147e-06 |
| ## | 2.5.1.141 | 0.9092807 | 6.602780e-06 |
| ## | 1.1.1.42 | 0.9088959 | 6.766824e-06 |
| ## | 1.5.1.2 | 0.9064491 | 7.890137e-06 |
| ## | 2.1.2.10 | 0.9064447 | 7.892303e-06 |
| ## | 2.7.6.3 | 0.9057532 | 8.236162e-06 |
| ## | 4.3.2.10 | 0.9052902 | 8.473193e-06 |
| ## | 5.4.99.9 | 0.9047627 | 8.750033e-06 |
| ## | 3.1.4.3 | 0.9039586 | 9.186293e-06 |
| ## | 3.5.2.17 | 0.9034366 | 9.478958e-06 |
| ## | 1.1.1.22 | 0.9032993 | 9.557197e-06 |
| ## | 2.7.7.9 | 0.9031109 | 9.665363e-06 |
| ## | 2.7.7.23 | 0.9019088 | 1.037974e-05 |
| ## | 2.3.1.157 | 0.9019088 | 1.037974e-05 |
| ## | 3.4.11.5 | 0.9011824 | 1.083200e-05 |
| ## | 1.17.3.2 | 0.9007740 | 1.109330e-05 |
| ## | 3.2.1.52 | 0.8998112 | 1.172981e-05 |
| ## | 4.1.3.27 | 0.8988437 | 1.239924e-05 |
| ## | 2.6.99.2 | 0.8982147 | 1.285107e-05 |
| ## | 6.3.4.20 | 0.8978936 | 1.308687e-05 |

| ## | 2.1.1.67 | 0.8972337 | 1.358257e-05 |
| --- | --- | --- | --- |
| ## | 6.1.1.5 | 0.8967932 | 1.392197e-05 |
| ## | 1.11.1.15 | 0.8948693 | 1.548680e-05 |
| ## | 2.7.4.6 | 0.8942450 | 1.602446e-05 |
| ## | 1.17.1.8 | 0.8942174 | 1.604863e-05 |
| ## | 6.3.2.8 | 0.8940269 | 1.621591e-05 |
| ## | 4.2.1.24 | 0.8939668 | 1.626897e-05 |
| ## | 2.7.7.3 | 0.8931189 | 1.703298e-05 |
| ## | 1.8.4.8 | 0.8927013 | 1.741996e-05 |
| ## | 2.7.9.1 | 0.8905010 | 1.957956e-05 |
| ## | 2.5.1.61 | 0.8892910 | 2.085748e-05 |
| ## | 4.1.1.31 | 0.8877515 | 2.258143e-05 |
| ## | 4.1.1.48 | 0.8873222 | 2.308229e-05 |
| ## | 2.3.1.30 | 0.8868618 | 2.362961e-05 |
| ## | 2.6.1.52 | 0.8863443 | 2.425741e-05 |
| ## | 5.3.1.6 | 0.8862894 | 2.432483e-05 |
| ## | 3.5.1.2 | 0.8860194 | 2.465850e-05 |
| ## | 5.1.1.1 | 0.8852860 | 2.558377e-05 |
| ## | 1.1.1.44 | 0.8838397 | 2.749176e-05 |
| ## | 2.2.1.6 | 0.8825700 | 2.926124e-05 |
| ## | 2.4.2.9 | 0.8804887 | 3.236241e-05 |
| ## | 3.6.1.1 | 0.8800728 | 3.301322e-05 |
| ## | 1.1.1.3 | 0.8797355 | 3.354877e-05 |
| ## | 2.7.1.49 | 0.8791729 | 3.445786e-05 |
| ## | 4.2.1.59 | 0.8784750 | 3.561349e-05 |
| ## | 3.6.1.54 | 0.8783186 | 3.587688e-05 |
| ## | 4.2.1.2 | 0.8756429 | 4.063270e-05 |
| ## | 4.1.2.25 | 0.8755219 | 4.085953e-05 |
| ## | 1.17.1.4 | 0.8721144 | 4.767932e-05 |
| ## | 3.5.4.9 | 0.8719009 | 4.813566e-05 |
| ## | 1.5.1.5 | 0.8719009 | 4.813566e-05 |
| ## | 2.1.1.107 | 0.8703975 | 5.145141e-05 |
| ## | 1.3.1.76 | 0.8701910 | 5.192103e-05 |
| ## | 4.99.1.4 | 0.8701447 | 5.202673e-05 |
| ## | 2.7.1.26 | 0.8690665 | 5.454012e-05 |
| ## | 3.1.3.7 | 0.8686039 | 5.564851e-05 |
| ## | 5.4.99.15 | 0.8676965 | 5.787579e-05 |
| ## | 2.7.7.4 | 0.8670069 | 5.961640e-05 |
| ## | 2.8.1.8 | 0.8669268 | 5.982137e-05 |
| ## | 3.6.1.40 | 0.8666102 | 6.063695e-05 |
| ## | 2.3.1.61 | 0.8661109 | 6.194163e-05 |
| ## | 3.6.1.27 | 0.8659624 | 6.233420e-05 |
| ## | 2.5.1.9 | 0.8657187 | 6.298250e-05 |
| ## | 2.1.3.3 | 0.8642144 | 6.710761e-05 |
| ## | 2.7.1.12 | 0.8640445 | 6.758684e-05 |
| ## | 2.5.1.16 | 0.8639107 | 6.796639e-05 |
| ## | 1.14.14.18 | 0.8631231 | 7.023551e-05 |
| ## | 3.2.1.141 | 0.8617774 | 7.425538e-05 |
| ## | 3.1.3.27 | 0.8615823 | 7.485341e-05 |
| ## | 2.5.1.54 | 0.8609719 | 7.674950e-05 |
| ## | 4.1.1.23 | 0.8607497 | 7.744942e-05 |
| ## | 1.8.1.4 | 0.8592307 | 8.237484e-05 |
| ## | 1.1.1.284 | 0.8589122 | 8.343908e-05 |
| ## | 4.1.3.30 | 0.8579562 | 8.670124e-05 |

| ## | 4.4.1.13 | 0.8567243 | 9.105695e-05 |
| --- | --- | --- | --- |
| ## | 3.4.19.13 | 0.8559801 | 9.377321e-05 |
| ## | 2.3.2.2 | 0.8559801 | 9.377321e-05 |
| ## | 3.5.4.25 | 0.8550695 | 9.718627e-05 |
| ## | 4.6.1.17 | 0.8546352 | 9.884927e-05 |
| ## | 2.6.1.16 | 0.8537568 | 1.022832e-04 |
| ## | 6.3.5.3 | 0.8535066 | 1.032789e-04 |
| ## | 5.3.1.23 | 0.8518771 | 1.099584e-04 |
| ## | 1.4.4.2 | 0.8498350 | 1.188201e-04 |
| ## | 2.7.7.13 | 0.8490590 | 1.223359e-04 |
| ## | 6.6.1.2 | 0.8466571 | 1.337569e-04 |
| ## | 6.3.4.2 | 0.8466145 | 1.339670e-04 |
| ## | 2.1.1.163 | 0.8465560 | 1.342560e-04 |
| ## | 1.8.1.2 | 0.8462481 | 1.357852e-04 |
| ## | 1.4.3.5 | 0.8450183 | 1.420345e-04 |
| ## | 2.7.2.3 | 0.8421560 | 1.574868e-04 |
| ## | 6.3.2.9 | 0.8400684 | 1.695938e-04 |
| ## | 3.5.2.9 | 0.8394292 | 1.734483e-04 |
| ## | 2.4.1.18 | 0.8393215 | 1.741044e-04 |
| ## | 6.3.2.10 | 0.8390618 | 1.756955e-04 |
| ## | 2.4.1.21 | 0.8377318 | 1.840287e-04 |
| ## | 1.2.4.2 | 0.8374694 | 1.857099e-04 |
| ## | 1.1.5.12 | 0.8370181 | 1.886307e-04 |
| ## | 2.3.1.1 | 0.8367858 | 1.901479e-04 |
| ## | 1.1.1.49 | 0.8361659 | 1.942471e-04 |
| ## | 2.7.7.60 | 0.8359193 | 1.958974e-04 |
| ## | 6.3.2.3 | 0.8303825 | 2.360462e-04 |
| ## | 1.1.1.219 | 0.8301221 | 2.380869e-04 |
| ## | 6.1.1.9 | 0.8300494 | 2.386586e-04 |
| ## | 2.5.1.78 | 0.8292773 | 2.448044e-04 |
| ## | 3.1.2.12 | 0.8288890 | 2.479428e-04 |
| ## | 3.5.4.3 | 0.8288620 | 2.481630e-04 |
| ## | 3.2.1.28 | 0.8283167 | 2.526282e-04 |
| ## | 6.3.5.2 | 0.8269163 | 2.643957e-04 |
| ## | 3.5.1.5 | 0.8257071 | 2.749086e-04 |
| ## | 2.5.1.6 | 0.8238401 | 2.918019e-04 |
| ## | 1.1.1.17 | 0.8208639 | 3.204570e-04 |
| ## | 3.3.1.1 | 0.8192451 | 3.369703e-04 |
| ## | 1.7.1.13 | 0.8191252 | 3.382199e-04 |
| ## | 1.2.1.27 | 0.8184618 | 3.452026e-04 |
| ## | 3.5.1.25 | 0.8175194 | 3.553221e-04 |
| ## | 4.2.1.20 | 0.8164324 | 3.672900e-04 |
| ## | 4.2.1.11 | 0.8164208 | 3.674199e-04 |
| ## | 1.3.98.3 | 0.8157022 | 3.755105e-04 |
| ## | 4.2.1.1 | 0.8149017 | 3.846924e-04 |
| ## | 2.6.1.1 | 0.8147887 | 3.860036e-04 |
| ## | 6.3.4.4 | 0.8128812 | 4.086739e-04 |
| ## | 1.17.7.3 | 0.8106442 | 4.366137e-04 |
| ## | 4.2.1.19 | 0.8105017 | 4.384452e-04 |
| ## | 3.5.1.1 | 0.8099954 | 4.449996e-04 |
| ## | 4.2.1.9 | 0.8098411 | 4.470122e-04 |
| ## | 4.1.1.8 | 0.8093887 | 4.529563e-04 |
| ## | 2.7.7.77 | 0.8093272 | 4.537695e-04 |
| ## | 2.7.1.33 | 0.8089194 | 4.591896e-04 |

| ## | 5.4.99.5 | 0.8086283 | 4.630913e-04 |
| --- | --- | --- | --- |
| ## | 2.3.1.8 | 0.8066902 | 4.897505e-04 |
| ## | 2.3.1.181 | 0.8064897 | 4.925761e-04 |
| ## | 1.1.1.267 | 0.8036879 | 5.334709e-04 |
| ## | 2.7.2.1 | 0.7999985 | 5.914362e-04 |
| ## | 5.3.1.9 | 0.7988435 | 6.105868e-04 |
| ## | 6.3.4.21 | 0.7984571 | 6.171035e-04 |
| ## | 2.5.1.7 | 0.7984464 | 6.172854e-04 |
| ## | 2.7.1.40 | 0.7962304 | 6.557531e-04 |
| ## | 6.1.1.2 | 0.7957277 | 6.647405e-04 |
| ## | 6.1.1.15 | 0.7936327 | 7.032702e-04 |
| ## | 3.5.1.10 | 0.7922359 | 7.299405e-04 |
| ## | 2.5.1.55 | 0.7889917 | 7.950148e-04 |
| ## | 3.6.1.23 | 0.7873746 | 8.291417e-04 |
| ## | 1.1.1.169 | 0.7836964 | 9.111448e-04 |
| ## | 1.5.1.20 | 0.7827247 | 9.338540e-04 |
| ## | 1.1.1.23 | 0.7816203 | 9.602148e-04 |
| ## | 3.1.3.11 | 0.7812522 | 9.691306e-04 |
| ## | 6.3.5.4 | 0.7793645 | 1.015904e-03 |
| ## | 2.3.1.129 | 0.7782954 | 1.043182e-03 |
| ## | 2.7.2.4 | 0.7762802 | 1.096190e-03 |
| ## | 6.3.5.5 | 0.7747885 | 1.136795e-03 |
| ## | 4.1.1.37 | 0.7732832 | 1.178979e-03 |
| ## | 2.3.1.37 | 0.7729228 | 1.189260e-03 |
| ## | 2.6.1.62 | 0.7725882 | 1.198871e-03 |
| ## | 4.4.1.5 | 0.7715501 | 1.229081e-03 |
| ## | 5.2.1.1 | 0.7709838 | 1.245815e-03 |
| ## | 1.1.1.205 | 0.7708348 | 1.250247e-03 |
| ## | 6.3.2.2 | 0.7675656 | 1.350720e-03 |
| ## | 1.1.1.262 | 0.7619818 | 1.537117e-03 |
| ## | 2.3.1.12 | 0.7593016 | 1.633566e-03 |
| ## | 1.13.11.53 | 0.7575164 | 1.700428e-03 |
| ## | 4.3.1.7 | 0.7563655 | 1.744676e-03 |
| ## | 1.1.5.3 | 0.7547051 | 1.810118e-03 |
| ## | 1.2.4.1 | 0.7543424 | 1.824666e-03 |
| ## | 6.1.1.6 | 0.7333488 | 2.839085e-03 |
| ## | 1.3.5.1 | 0.7327526 | 2.873289e-03 |
| ## | 6.3.1.2 | 0.7279176 | 3.162846e-03 |
| ## | 3.4.16.4 | 0.7244912 | 3.381602e-03 |
| ## | 2.7.8.7 | 0.7218074 | 3.561126e-03 |
| ## | 2.1.2.1 | 0.7205702 | 3.646358e-03 |
| ## | 6.3.3.3 | 0.7169965 | 3.901603e-03 |
| ## | 6.3.5.7 | 0.7165649 | 3.933356e-03 |
| ## | 4.2.1.33 | 0.7131785 | 4.189583e-03 |
| ## | 1.2.1.38 | 0.7050123 | 4.861429e-03 |
| ## | 1.11.1.6 | 0.7023126 | 5.101132e-03 |
| ## | 3.1.3.2 | 0.7016365 | 5.162587e-03 |
| ## | 4.1.1.36 | 0.6943398 | 5.863294e-03 |
| ## | 6.1.1.14 | 0.6874159 | 6.594705e-03 |
| ## | 1.1.5.4 | 0.6832041 | 7.073121e-03 |
| ## | 6.3.4.5 | 0.6807649 | 7.362261e-03 |
| ## | 5.4.99.16 | 0.6776411 | 7.745844e-03 |
| ## | 1.2.1.12 | 0.6743739 | 8.163436e-03 |
| ## | 3.1.1.31 | 0.6701714 | 8.725952e-03 |

| ## | 6.1.1.20 | 0.6695099 | 8.817155e-03 |
| --- | --- | --- | --- |
| ## | 6.3.4.13 | 0.6683897 | 8.973276e-03 |
| ## | 2.7.1.158 | -0.6617301 | 9.946029e-03 |
| ## | 2.4.2.22 | -0.6693914 | 8.833569e-03 |
| ## | 2.7.1.58 | -0.6711147 | 8.597154e-03 |
| ## | 1.1.1.350 | -0.6800192 | 7.452461e-03 |
| ## | 2.4.1.25 | -0.6826380 | 7.139426e-03 |
| ## | 3.4.11.23 | -0.6854617 | 6.813445e-03 |
| ## | 6.4.1.1 | -0.6861312 | 6.737884e-03 |
| ## | 2.4.1.12 | -0.6866282 | 6.682209e-03 |
| ## | 5.1.3.3 | -0.6891285 | 6.407528e-03 |
| ## | 4.1.99.19 | -0.6899289 | 6.321486e-03 |
| ## | 4.1.2.4 | -0.6962187 | 5.676173e-03 |
| ## | 1.14.11.8 | -0.6971664 | 5.583579e-03 |
| ## | 4.1.3.34 | -0.6974513 | 5.555969e-03 |
| ## | 2.7.1.6 | -0.6985504 | 5.450468e-03 |
| ## | 1.6.99.3 | -0.7009672 | 5.223989e-03 |
| ## | 2.7.7.12 | -0.7050957 | 4.854166e-03 |
| ## | 7.6.2.7 | -0.7061177 | 4.765859e-03 |
| ## | 1.17.1.9 | -0.7080089 | 4.605777e-03 |
| ## | 5.1.3.9 | -0.7094652 | 4.485395e-03 |
| ## | 1.5.99.12 | -0.7098228 | 4.456216e-03 |
| ## | 4.3.1.3 | -0.7126710 | 4.229083e-03 |
| ## | 2.1.2.13 | -0.7132561 | 4.183571e-03 |
| ## | 1.1.1.305 | -0.7132561 | 4.183571e-03 |
| ## | 3.6.1.29 | -0.7139266 | 4.131885e-03 |
| ## | 1.1.1.330 | -0.7142998 | 4.103335e-03 |
| ## | 4.2.1.104 | -0.7150217 | 4.048545e-03 |
| ## | 5.5.1.19 | -0.7168853 | 3.909764e-03 |
| ## | 2.4.1.16 | -0.7170493 | 3.897735e-03 |
| ## | 2.1.1.22 | -0.7182100 | 3.813411e-03 |
| ## | 1.1.1.127 | -0.7261702 | 3.272968e-03 |
| ## | 2.7.1.137 | -0.7300981 | 3.029551e-03 |
| ## | 3.7.1.12 | -0.7315975 | 2.940484e-03 |
| ## | 2.4.1.255 | -0.7392540 | 2.517340e-03 |
| ## | 5.3.2.3 | -0.7394074 | 2.509386e-03 |
| ## | 4.1.1.68 | -0.7394721 | 2.506034e-03 |
| ## | 3.2.2.5 | -0.7434019 | 2.309119e-03 |
| ## | 3.1.3.99 | -0.7465959 | 2.158265e-03 |
| ## | 4.3.3.6 | -0.7470914 | 2.135581e-03 |
| ## | 3.2.1.23 | -0.7497281 | 2.018016e-03 |
| ## | 4.4.1.21 | -0.7518611 | 1.926721e-03 |
| ## | 3.5.4.6 | -0.7539130 | 1.842011e-03 |
| ## | 1.1.1.336 | -0.7565415 | 1.737850e-03 |
| ## | 4.2.1.17 | -0.7566341 | 1.734269e-03 |
| ## | 1.1.1.35 | -0.7566341 | 1.734269e-03 |
| ## | 2.7.1.48 | -0.7599762 | 1.608848e-03 |
| ## | 1.1.1.93 | -0.7616576 | 1.548538e-03 |
| ## | 1.1.1.83 | -0.7616576 | 1.548538e-03 |
| ## | 1.13.11.1 | -0.7619233 | 1.539174e-03 |
| ## | 4.2.1.42 | -0.7620773 | 1.533768e-03 |
| ## | 2.7.1.198 | -0.7622985 | 1.526028e-03 |
| ## | 2.1.1.14 | -0.7637617 | 1.475609e-03 |
| ## | 1.3.1.12 | -0.7641182 | 1.463530e-03 |

| ## | 3.2.2.9 | -0.7644533 | 1.452246e-03 |
| --- | --- | --- | --- |
| ## | 4.2.1.48 | -0.7658521 | 1.405890e-03 |
| ## | 3.5.1.54 | -0.7663452 | 1.389833e-03 |
| ## | 4.2.1.147 | -0.7672295 | 1.361405e-03 |
| ## | 4.1.2.43 | -0.7672295 | 1.361405e-03 |
| ## | 5.5.1.4 | -0.7674151 | 1.355498e-03 |
| ## | 6.1.1.22 | -0.7675854 | 1.350094e-03 |
| ## | 5.3.1.8 | -0.7681970 | 1.330833e-03 |
| ## | 5.3.1.26 | -0.7721951 | 1.210242e-03 |
| ## | 4.1.2.13 | -0.7725220 | 1.200779e-03 |
| ## | 2.4.1.256 | -0.7742137 | 1.152759e-03 |
| ## | 1.3.8.8 | -0.7744308 | 1.146708e-03 |
| ## | 1.3.8.7 | -0.7744308 | 1.146708e-03 |
| ## | 1.13.11.15 | -0.7755360 | 1.116299e-03 |
| ## | 3.7.1.22 | -0.7763224 | 1.095059e-03 |
| ## | 2.8.1.4 | -0.7773662 | 1.067365e-03 |
| ## | 4.1.99.1 | -0.7793964 | 1.015100e-03 |
| ## | 4.2.1.70 | -0.7802344 | 9.941307e-04 |
| ## | 3.1.4.16 | -0.7843889 | 8.952297e-04 |
| ## | 3.1.3.6 | -0.7843889 | 8.952297e-04 |
| ## | 1.13.11.11 | -0.7850804 | 8.795609e-04 |
| ## | 2.7.1.208 | -0.7858557 | 8.622556e-04 |
| ## | 1.13.11.20 | -0.7863032 | 8.523911e-04 |
| ## | 4.1.1.22 | -0.7864148 | 8.499468e-04 |
| ## | 3.5.2.7 | -0.7869195 | 8.389564e-04 |
| ## | 2.7.1.21 | -0.7879369 | 8.171445e-04 |
| ## | 1.1.98.6 | -0.7895945 | 7.825856e-04 |
| ## | 1.5.1.7 | -0.7904803 | 7.646026e-04 |
| ## | 2.7.7.53 | -0.7906355 | 7.614855e-04 |
| ## | 2.7.7.5 | -0.7906355 | 7.614855e-04 |
| ## | 6.4.1.2 | -0.7938017 | 7.000983e-04 |
| ## | 4.2.1.68 | -0.7940013 | 6.963645e-04 |
| ## | 1.2.1.50 | -0.7943792 | 6.893414e-04 |
| ## | 6.2.1.20 | -0.7952892 | 6.726616e-04 |
| ## | 2.3.1.40 | -0.7952892 | 6.726616e-04 |
| ## | 2.7.1.17 | -0.7953057 | 6.723631e-04 |
| ## | 2.7.1.16 | -0.7970763 | 6.408491e-04 |
| ## | 4.2.1.44 | -0.7977676 | 6.288707e-04 |
| ## | 2.6.1.85 | -0.7995484 | 5.988421e-04 |
| ## | 3.5.99.7 | -0.8032069 | 5.407578e-04 |
| ## | 1.13.11.3 | -0.8042388 | 5.252217e-04 |
| ## | 2.3.1.199 | -0.8085463 | 4.641946e-04 |
| ## | 1.4.1.21 | -0.8102480 | 4.417189e-04 |
| ## | 1.11.1.9 | -0.8108850 | 4.335353e-04 |
| ## | 3.1.3.4 | -0.8111591 | 4.300505e-04 |
| ## | 1.5.1.36 | -0.8114309 | 4.266183e-04 |
| ## | 2.6.1.19 | -0.8118645 | 4.211882e-04 |
| ## | 5.3.1.30 | -0.8136581 | 3.993144e-04 |
| ## | 5.3.1.14 | -0.8137632 | 3.980622e-04 |
| ## | 5.3.3.4 | -0.8141149 | 3.938928e-04 |
| ## | 4.2.1.22 | -0.8142150 | 3.927123e-04 |
| ## | 4.1.3.17 | -0.8145779 | 3.884573e-04 |
| ## | 2.7.1.107 | -0.8146015 | 3.881818e-04 |
| ## | 4.4.1.17 | -0.8150889 | 3.825298e-04 |

| ## | 2.7.9.3 | -0.8153774 | 3.792145e-04 |
| --- | --- | --- | --- |
| ## | 4.3.1.17 | -0.8182488 | 3.474702e-04 |
| ## | 1.7.99.1 | -0.8197815 | 3.314242e-04 |
| ## | 2.7.1.197 | -0.8208583 | 3.205126e-04 |
| ## | 2.7.1.100 | -0.8210390 | 3.187105e-04 |
| ## | 2.7.1.166 | -0.8213398 | 3.157284e-04 |
| ## | 4.1.1.74 | -0.8221591 | 3.077198e-04 |
| ## | 3.1.2.14 | -0.8233739 | 2.961485e-04 |
| ## | 4.1.2.20 | -0.8240258 | 2.900853e-04 |
| ## | 2.3.1.16 | -0.8249856 | 2.813409e-04 |
| ## | 4.7.1.1 | -0.8253468 | 2.781057e-04 |
| ## | 5.3.3.14 | -0.8269569 | 2.640481e-04 |
| ## | 2.7.9.2 | -0.8289650 | 2.473261e-04 |
| ## | 1.14.13.9 | -0.8292607 | 2.449376e-04 |
| ## | 5.3.3.2 | -0.8304928 | 2.351863e-04 |
| ## | 2.3.1.5 | -0.8310005 | 2.312600e-04 |
| ## | 1.5.1.3 | -0.8313708 | 2.284303e-04 |
| ## | 3.2.1.1 | -0.8315473 | 2.270920e-04 |
| ## | 1.14.14.17 | -0.8318461 | 2.248395e-04 |
| ## | 3.2.1.39 | -0.8319266 | 2.242355e-04 |
| ## | 1.1.1.261 | -0.8332954 | 2.141705e-04 |
| ## | 2.7.1.29 | -0.8333523 | 2.137598e-04 |
| ## | 2.7.1.28 | -0.8333523 | 2.137598e-04 |
| ## | 2.6.1.13 | -0.8334391 | 2.131351e-04 |
| ## | 3.2.1.55 | -0.8352060 | 2.007351e-04 |
| ## | 5.1.3.14 | -0.8361111 | 1.946127e-04 |
| ## | 4.1.1.15 | -0.8368779 | 1.895454e-04 |
| ## | 1.1.1.38 | -0.8370789 | 1.882347e-04 |
| ## | 4.3.2.3 | -0.8379229 | 1.828118e-04 |
| ## | 1.13.11.63 | -0.8379237 | 1.828070e-04 |
| ## | 2.7.1.201 | -0.8390383 | 1.758401e-04 |
| ## | 2.6.1.82 | -0.8394842 | 1.731139e-04 |
| ## | 2.7.8.8 | -0.8396468 | 1.721280e-04 |
| ## | 3.1.3.89 | -0.8406019 | 1.664307e-04 |
| ## | 7.1.2.1 | -0.8407890 | 1.653326e-04 |
| ## | 3.5.4.5 | -0.8411960 | 1.629639e-04 |
| ## | 2.7.1.56 | -0.8412299 | 1.627683e-04 |
| ## | 2.7.11.24 | -0.8421313 | 1.576255e-04 |
| ## | 3.11.1.1 | -0.8425604 | 1.552243e-04 |
| ## | 1.14.99.46 | -0.8427193 | 1.543426e-04 |
| ## | 2.1.1.45 | -0.8431840 | 1.517875e-04 |
| ## | 2.6.1.87 | -0.8435988 | 1.495359e-04 |
| ## | 5.1.3.4 | -0.8445103 | 1.446832e-04 |
| ## | 3.5.3.26 | -0.8456434 | 1.388296e-04 |
| ## | 4.3.1.1 | -0.8457100 | 1.384916e-04 |
| ## | 2.7.4.24 | -0.8459458 | 1.373005e-04 |
| ## | 2.7.4.21 | -0.8459458 | 1.373005e-04 |
| ## | 4.3.1.18 | -0.8467532 | 1.332841e-04 |
| ## | 3.1.3.70 | -0.8467672 | 1.332150e-04 |
| ## | 4.2.1.84 | -0.8499151 | 1.184619e-04 |
| ## | 1.8.1.7 | -0.8502411 | 1.170131e-04 |
| ## | 1.13.11.57 | -0.8514878 | 1.116048e-04 |
| ## | 3.5.3.1 | -0.8529220 | 1.056362e-04 |
| ## | 1.4.1.14 | -0.8544773 | 9.945960e-05 |

## 2.3.1.242 -0.8550199 9.737483e-05

## 4.2.1.49 -0.8558198 9.436686e-05

## 5.1.3.22 -0.8566551 9.130679e-05

## 4.1.1.21 -0.8575964 8.795543e-05

## [ reached getOption("max.print") -- omitted 128 rows ] ##

##

## $Dim.2

## $Dim.2$quanti

## correlation p.value

| ## | 3.5.3.6 | 0.8601962 | 7.921576e-05 |
| --- | --- | --- | --- |
| ## | 1.7.1.7 | 0.8535198 | 1.032265e-04 |
| ## | 5.3.1.5 | 0.7895116 | 7.842840e-04 |
| ## | 4.2.1.40 | 0.7893065 | 7.885040e-04 |
| ## | 4.2.1.79 | 0.7824831 | 9.395713e-04 |
| ## | 2.4.2.8 | 0.7714821 | 1.231080e-03 |
| ## | 1.14.14.9 | 0.7691478 | 1.301324e-03 |
| ## | 2.2.1.9 | 0.7670449 | 1.367299e-03 |
| ## | 4.1.2.40 | 0.7575038 | 1.700910e-03 |
| ## | 2.7.1.121 | 0.7374315 | 2.613396e-03 |
| ## | 5.4.2.11 | 0.7174603 | 3.867707e-03 |
| ## | 5.3.1.12 | 0.7070682 | 4.684872e-03 |
| ## | 6.3.1.20 | 0.7048206 | 4.878155e-03 |
| ## | 4.2.99.20 | 0.7025096 | 5.083333e-03 |
| ## | 5.3.1.4 | 0.6999280 | 5.320455e-03 |
| ## | 5.1.2.1 | 0.6997837 | 5.333960e-03 |
| ## | 2.7.1.5 | 0.6981454 | 5.489162e-03 |
| ## | 4.1.2.4 | 0.6906993 | 6.239508e-03 |
| ## | 2.3.3.10 | 0.6853644 | 6.824483e-03 |
| ## | 6.3.1.1 | 0.6812364 | 7.305674e-03 |
| ## | 4.2.1.28 | 0.6803264 | 7.415197e-03 |
| ## | 4.1.3.34 | 0.6785723 | 7.629915e-03 |
| ## | 6.2.1.26 | 0.6782364 | 7.671588e-03 |
| ## | 4.1.3.36 | 0.6766450 | 7.871369e-03 |
| ## | 3.2.1.93 | 0.6745025 | 8.146676e-03 |
| ## | 4.2.1.82 | 0.6694270 | 8.828636e-03 |
| ## | 2.7.1.6 | 0.6658784 | 9.331047e-03 |
| ## |  |  |  |
| ## |  |  |  |

## $Dim.3

## $Dim.3$quanti

## correlation p.value

| ## | 1.4.3.16 | 0.8129398 | 0.0004079614 |
| --- | --- | --- | --- |
| ## | 3.5.1.9 | 0.7319602 | 0.0029192503 |
| ## | 1.14.14.5 | 0.7213816 | 0.0035902819 |
| ## | 1.7.3.3 | -0.6715300 | 0.0085409128 |
| ## | 4.1.1.33 | -0.6808482 | 0.0073522390 |
| ## | 3.5.2.5 | -0.7009573 | 0.0052248979 |
| ## | 2.7.1.36 | -0.7012513 | 0.0051978551 |
| ## | 2.7.4.2 | -0.7036652 | 0.0049799129 |
| ## | 1.1.1.34 | -0.7584431 | 0.0016654531 |
| ## | 1.3.3.4 | -0.8287927 | 0.0002487262 |

## $Dim.4

## $Dim.4$quanti

## correlation p.value

| ## | 1.2.1.10 | 0.8768865 | 3.836207e-05 |
| --- | --- | --- | --- |
| ## | 4.1.3.39 | 0.8226401 | 3.030952e-04 |
| ## | 3.5.1.53 | 0.7652214 | 1.426644e-03 |
| ## | 3.1.3.8 | 0.7541918 | 1.830735e-03 |
| ## | 3.4.24.84 | 0.7532285 | 1.869936e-03 |
| ## | 4.1.1.96 | 0.7323790 | 2.894887e-03 |
| ## | 3.5.3.12 | 0.7306520 | 2.996403e-03 |
| ## | 2.3.1.222 | 0.6921813 | 6.084153e-03 |
| ## | 5.5.1.6 | 0.6694845 | 8.820669e-03 |
| ## | 7.2.4.2 | 0.6693768 | 8.835587e-03 |
| ## | 2.7.1.168 | 0.6654312 | 9.395892e-03 |
| ## | 2.4.1.131 | -0.7939307 | 6.976837e-04 |
| ## |  |  |  |
| ## |  |  |  |

## $Dim.5

## $Dim.5$quanti

## correlation p.value

| ## | 1.1.1.304 0.7941401 0.0006937783 | | |
| --- | --- | --- | --- |
| ## | 6.2.1.30 0.7922376 0.0007299072 | | |
| ## | 2.7.2.2 0.7623588 0.0015239238 | | |
| ## | 5.3.3.18 0.7546109 0.0018138865 | | |
| ## | 1.14.13.149 0.7284427 0.0031303363 | | |
| ## | 2.3.1.184 0.7233703 0.0034556960 | | |
| ## | 3.3.2.12 0.7221321 0.0035390130 | | |
| ## | 1.2.1.91 0.7221321 0.0035390130 | | |
| ## | 7.1.1.2 0.7066539 0.0047200418 | | |
| ## | 2.1.1.152 0.6975600 0.0055454690 | | |
| ## | 3.5.4.44 0.6901872 0.0062939083 | | |
| ## | 3.5.1.125 0.6901872 0.0062939083 | | |
| ## | 1.1.1.271 -0.6711547 0.0085917231 | | |
| ## |  | | |
| ## |  | | |
| ## | $Dim.6 | | |
| ## | $Dim.6$quanti | | |
| ## | correlation p.value | | |
| ## | 2.4.1.11 0.7699630 0.001276440 | | |
| ## | 3.1.4.4 | 0.7554848 | 0.001779147 |
| ## | 3.2.1.31 | 0.6671284 | 0.009151613 |
| ## | 1.14.18.1 | 0.6618508 | 0.009927715 |
| ## |  |  |  |
| ## |  |  |  |
| ## | $Dim.8 |  |  |
| ## | NULL |  |  |
| ## |  | | |
| ## | $Dim.9 | | |
| ## | $Dim.9$quanti | | |
| ## | correlation p.value | | |
| ## | 2.3.1.41 -0.7042035 0.004932296 | | |
| ## | 2.7.7.81 -0.7113610 0.004332393 | | |

## $Dim.10

## $Dim.10$quanti

## correlation p.value ## 1.7.2.4 -0.8117398 4.227441e-04

## 3.5.99.6 -0.8632770 6.978751e-05 ##

##

## $Dim.12 ## NULL ##

## [[11]] ## NULL ##

## [[12]]

## [[12]]$quanti

## correlation p.value ## 5.4.4.4 0.7680254 0.001336213

## 4.2.1.127 0.7680254 0.001336213

##plotting contributuin of each PCA vector ... 1 captures almost 80% of the variance

**fviz_eig**(res.PCA)

## Scree plot

40

Percentage of explained variances

20

0

1 2 3 4 5 6 7 8 9 10

Dimensions

##Biplot - visualising general patterns

**fviz_pca_biplot**(res.PCA,

col.var = "#4C9900", *# Variables color*

alpha.var=.1,

col.ind = "#696969" *# Individuals color*

)

## PCA − Biplot


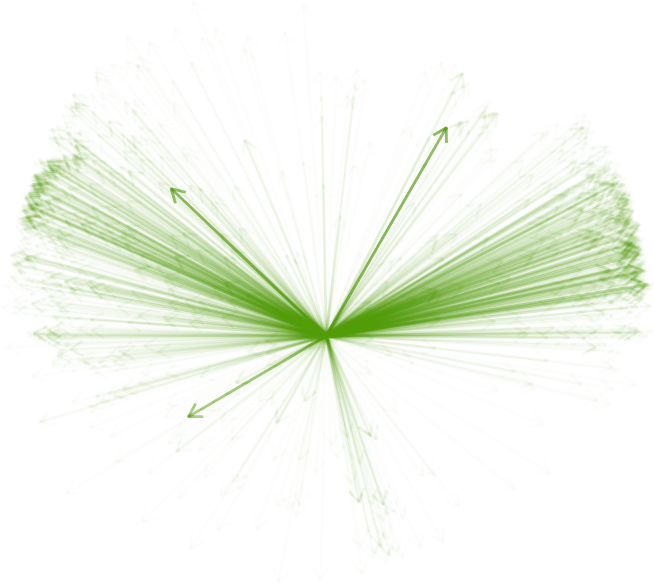


For144

3.5.3.6 1.7.1.7

Hib0

Hib24

424.5.1.571..5.3.3.232...4.13.714.4.42.41.1.2.125.1929.7.2.10.1212.12..31..27..38.6.21660..33653....1121....1312.0321636

24.4.1.52.2.382.44..1.2012...5.11.4.944..1024.1.9.79

44.2.422.1.7.17.1..142..3.32.32243.725.14..3110123

21212121241.22.1.3.12.4.7.42.1627.2.4.7.254.263.6131.12.32.7.2.7.14..1.2.1.131..13.11.715.25.4.1.6712.11.**.**2.413.1.841173.35.06.96.1584.012.25357465**.**.8252.6.11.02.74.832.1428..143212.7.2141..4..572912..1.1.1712.13.22.1.17...1.383731357.61

1232211.12..32.5.1..1.173.73247.2.7.4**.**14.1....8.11..1721.7.57.1**.**.23.1..1.88.5.129.295.1851095.533494.7321.32912..17.1412.2...931131.3...1251..811.52...125.34.1.9.292.1.1.37

2.4711421...2.411.1.1.7332.7..1..522.2**.**21..1.0.412.**.**314221.**.**305193160.4913547.75781..2.21.4.2**.**.32.1592.8.19.21.02.686 34

125.15...23157713.33.3..471.1.31.1.21..13..114.2.151313.13..6212.9160.9241..208961.227.791.61631.133.1.5.1.27851.9.29119416..1324067.3.2. 1.1. 6.33..412221..4212.3.5141.12413417.313..4271...322.3..2.65.1.2166.1731.32..233.1..7.1.24.121.15915.315010.4.2.429570.814255733.10752.7..13914.1732.4211651.2.1.5.1.7.13131.36.3...2.21.16.1.4.165..4.291825

41324.5..21.2.7.784.3...197.1.2.**1**854..47.2.4.7448693.1.12.259

63.11.52..11.12..12.31.2.3.2965.50.1.74

4.62 1.34 6

31365....63.16.34....11.1.1512....11.23.1862417739. . .169

.

1..2.419.179913.46 1. 43. 5.34.80

3.71.1.31.2.1.237.15.2.5.

4.2.3.54666..431...312.3.1..221452.1.2.2..5.1412.32.5.42.15.13.323.2.3.7..1.7.1.1.27513..1.114.13**.**251.671.8.1..2.17.372120.1.212.1..15147761**.**4..18..21311.63.5929373704278

3115.3.112...5.391..91.8682.71.1.23.61263.6731...3561.3.73.1..531.23427.3.2172267

5.36.1615242.3.3.236..42321.41.22.115.5.24523..17278.91.1..1.61.3.71429.1121.1.1..2.2.1.1.7132353.921328.4..0.59362394

4

1 3.42. 5.1. . 1475

13.3312332.33.1.4.162621.1.57.215.31..31.4131.3.318133.9.423.53.120.36..3.13211.79.3639171.218151911102.4.702.4540.15.1125....8.4..111.214...1.15.742..1742...351.0.29.91..122461.31.61..3.58.1.52..3242..5241**.**11.2.29.1..1074.21212.24322.212..11.721.8..3.1**.**43..551.7.34.135.1841..14.0.910..112.1.1.1..15..14219 3.31..3342.3.28.29292115.9793710372F2544H9oirb112404

1223423.233432.13.**.**4.47.1..1.6.11..5.56143.1.711.2..4.1...4**.**1.33.3.1.1211.22.54**.**41.6..1.1.11.21..5.4.44..5.219.423.17.121.2139.451.01551..6178.22.23.701.5**1**78**.**..71.1511.3..81.1975.2873.2.1.1324.2.1.47

15.421.33**.**7.4111.541..3373..41.3724974.**.**149.193182..97.1.1.122.3...133.249....1.39271....1128..064.8222

213432....72.2442H5..23..1..1.7.31.11i.1.72b175..11..1.1.2741..43.1727103.481.21827.2.7.1.2.1.1712119..0.3215.981..32.36512..6.7.7514.1218.142.11..13543...161..41.45

13.412.221413.21.1.118..92.125.216...1..541..7.316163.121.9..2.11.194.91.4812.3.4.32.6321.6261**.**212352.3.727228.4.21..3.25.10.32

1.2.1.131.1.1.13

322.4161.1.26..3.231.3124.2.22191.1.6.1.3175.5.2.241.25.461.3.1261.1..1.6..1771.2..4374.1.1219.2..1..3435**.**9.925328531612.4..3820187.2127298208

263.3..24432212..1322**.**2..21.436**.**.5.1.42..2.1151...1.24..6..1403.46..1..55.1.41926423..4..1561.21911816.0.5.31132.8.73**.**4141..51...247..13..31.3.11.4.85 32..62..14143.1.16.12.5.71.1.1.2.1441775

4.4.1.149.22..111...131.6.112.925.1413...2.2.128.114271..2.2452221..3.2.2.117.5141.122711..5**.**3.1.1.731.3.329.8.3.271711.156.1...1.11.0..121.13.115.2.641916.3.2132..5021483.887.2931754855797

67.2.31..5.422...1.132.5.92371.414..18.3.1212..1.2.41.1.31.1.1..2.27125.31.13..13..1222.12.4612141135.7..1.01544.2..1.21781571..73122.8121**.**12974.421.9185.745308

2.333.4233.6.24.26.391..4.421.1.753..3..315.43.821..141**.**.491.223291.877161...5.331.5.4.13847.5.141.4432.12.4..41.12.33.1713.11.245.12.3.37..5.37.91.2.49

22131321.2.**.**.44.721127..7**.**77**.**13..113.17**1**1..1.1.1.4.**.**51..31112813953..14.2.0..15.73218.94.41964.479.930791

13.15.1.31..1.46.11.4.29.3.3.292.63.2...1302.16...1.931.971315..1321.2.6. 1.

17 1.14.1384..5.11.9..129..416.10

.4.1.12.3.112..42..3263..11121..214.13.5..724.147..1.63.172.57.9.12118.48318

43..62651...11.191.2.911.3311..0..2.3233.516212204...14.39..124.1.31.71.762.7.781.5.5..4.113.4.1**.**7636.731..1**.**91..9.52**.**41.3.66.25.1571.6251068

1.13.176.523.2553..2.1.21.57.442.12..811.11255728.3.7..3..2252.3H7..1.1.1490.74.11.9.5..11i1.4.0**.**8b3.1148....2.1.1730.92.49...121250321120

**3.**524..32.12...66.31.1.1.20392132323.121.5.3.1.17515.8.5441..211.15.3211.1.124876.39734.170324.18.1.12448.91.96.917 3.6H.1ib.796

512.2.511..1441**.**114..12121541..959.363.210.73.12 1.241.1.4245

64.2.1.108

1.12.1.4.8.16.1245..231..415 16.1

2.61.32.49132..61..13..534

Fo3r.24.1.12.24145.2.612..2324.1.3044.271.231122..75714.2.842.3274

442..1.23..2.131..1**.**18143343.5.1.139..5.15..

6.3.453.. 1.3.8..1.1..1.480

1..364.6..213132..3.6.5141..1.341..271

1.13.11.111.1.14.16.212.9.547...1.5371...14.1.18.1..1.1643424.**.**.1.61171..3..13.**1**31 86.14.17131313879.215H.3iF.b8o7r272

3.2.13.2.0.2356..391.8..13..1.8556.31.3.81.1.3.220..1..371...7583.23.253..11..25.35

2.1.2.11

For4318.2437.15.1.3.1.1.22

5.4.2.2

2.4.99.16

5.4.99.16

4.2.1.946.22..21..61 17 43.5.24.1.1.21.949.1.08.946

1.4.1.2 2.471621...1..1412..2431.1.4.6398129.83.49.6..24814..5.1509.2

5.41.21.3812.1..13.1.2.114..3..321.146.7.210

11.5

For96

For0

20

10

0

Dim2 (12%)

−10

−40 −20 0 20

### Dim1 (55.2%)

##Graph of variables. Positive correlated variables point to the same side of the plot. Negative correl

**fviz_pca_var**(res.PCA,

col.var = "contrib", *# Color by contributions to the PC*

alpha.var = .5,

gradient.cols = **c**("#00AFBB", "#E7B800", "#FC4E07"),

label="none",

repel = TRUE *# Avoid text overlapping*

)

1.0

0.5

0.0

Dim2 (12%)

−0.5

−1.0

## Variables − PCA

|  |  |  |  |  |  |  |  |  |  |
| --- | --- | --- | --- | --- | --- | --- | --- | --- | --- |
|  |  |  |  |  |  |  |  |  |  |
|  |  |  |  |  |  |  |  |  |  |
|  |  |  |  |  |  |  |  |  |  |
|  |  |  |  |  |  |  |  |  |  |
|  |  |  |  |  |  |  |  |  |  |
|  |  |  |  |  |  |  |  |  |  |
|  |  |  |  |  |  |  |  |  |  |
|  |  |  |  |  |  |  |  |  |  |
|  |  |  |  |  |  |  |  |  |  |


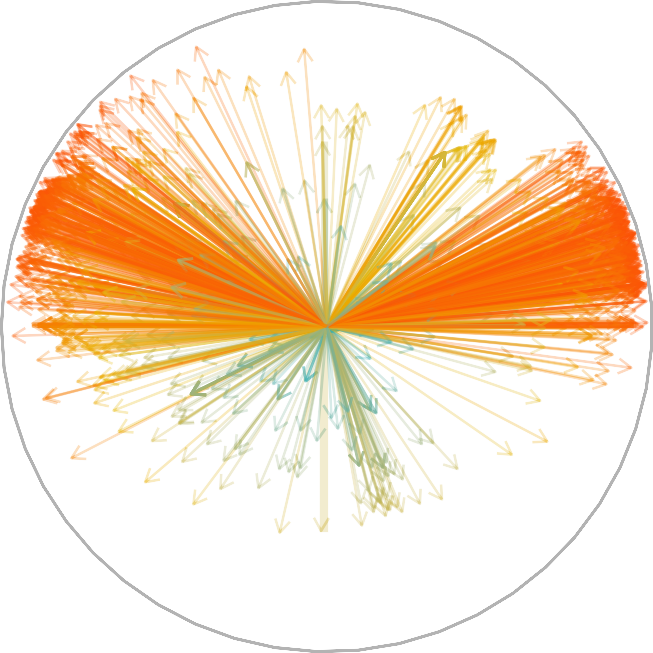
−1.0 −0.5 0.0 0.5 1.0

### Dim1 (55.2%)

### contrib

0.12


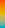


0.08

0.04

*#Graph of individuals. Individuals with a similar profile are grouped together*

**fviz_pca_ind**(res.PCA,

col.ind = "cos2", *# Color by the quality of representation*

gradient.cols = **c**("#00AFBB", "#E7B800", "#FC4E07"),

repel = TRUE *# Avoid text overlapping*

)

20

10

Dim2 (12%)

0

−10

## Individuals − PCA

|  |  |  |  |  |  |  | For144 |  |
| --- | --- | --- | --- | --- | --- | --- | --- | --- |
|  | Hib0 |  |  |  |  |  |  |  |
|  |  | Hib24 |  |  |  |  |  |  |
|  |  | Hi | b48 |  |  |  | Hib14 | 4 |
|  |  |  |  |  |  | Hib | For120 120 |  |
|  |  |  |  |  | Hib96 |  |  |  |
|  | For0 | For | 24  For4 | 8 | Hib7 | 2 |  |  |
|  |  |  |  |  |  | For72 |  |  |
|  |  |  |  |  | For96 |  |  |  |

−40 −20 0 20

### Dim1 (55.2%)

### cos2

0.8


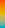


0.6

0.4

varieties <- **rownames**(dados2)

varieties[**grep**(pattern = "For", x = varieties)] <- "Forastero" varieties[**grep**(pattern = "Hib", x = varieties)] <- "Hybrid" varieties <- **as.factor**(varieties)

**fviz_pca_ind**(res.PCA,

col.ind = varieties, *# color by groups*

palette = **c**("#FF3333", "#4C9900"),

addEllipses = TRUE, *# Concentration ellipses*

ellipse.type = "confidence", legend.title = "Groups", repel = TRUE

)

## Individuals − PCA


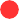


For144

Hib24

Hib0

Hib48

Hib144 For120

Hib120

For24

For48

Hib72

For72

For96

For0

Hib96

20

10

### Groups

Dim2 (12%)

Forastero

a

a

0 Hybrid

−10

−40 −20 0 20

### Dim1 (55.2%)

###Trying to group variables using NMDS, it can be easier to see dados2 <- **decostand**(dados2, method = "hel")

NMDS <- **metaMDS**(dados2, k=2, trymax = 10000, trace = F)

##MULTIPLE STARTS

sol1 <- **metaMDS**(dados2, k=2, trymax = 10000, trace = F)

**for**(i **in** 1**:**100){

solu <- **paste**("sol",i,sep="")

sol_new <- **metaMDS**(dados2, previous.best=**eval**(**as.name**(solu)), k=2, trace=F, trymax=10000)

**assign**(**paste**("sol",i**+**1,sep=""), sol_new)

}

(NMDS <- sol_new)

##

## Call:

## metaMDS(comm = dados2, k = 2, trymax = 10000, trace = F, previous.best = eval(as.name(solu))) ##

## global Multidimensional Scaling using monoMDS ##

## Data: dados2 ## Distance: bray ##

## Dimensions: 2

## Stress: 0.03089656

## Stress type 1, weak ties

## Two convergent solutions found after 2020 tries

## Scaling: centring, PC rotation, halfchange scaling ## Species: expanded scores based on 'dados2'

axis.text.y=**e**

beepr**::beep**(2)

**library**(data.table)

*#df <- data.frame(mds.jac$points[,1], mds.jac$points[,2], data$Total_AGB, data[,5])* df <- **data.table**(mds1=NMDS**$**points[,1], mds2=NMDS**$**points[,2], groups=varieties) hulls <- df[,.SD[**chull**(mds1, mds2)], by=groups]

**ggplot**(df, **aes**(x=mds1, y=mds2))**+**

**geom_polygon**(data=hulls, **aes**(colour=groups), linetype=3, size=2, alpha=0)**+ geom_point**(shape=21, **aes**(fill=groups), alpha=.9, size=4)**+ scale_fill_manual**(values=**c**("#FF3333", "#4C9900"))**+ scale_colour_manual**(values=**c**("#FF3333", "#4C9900"))**+**

**theme_classic**()**+**

**theme**(legend.position="bottom", legend.title=**element_blank**())**+ scale_x_continuous**("NMDS1")**+**

**scale_y_continuous**("NMDS2")**+**

**theme**(text = **element_text**(size=20),axis.text.x = **element_text**(vjust=1, colour="black"),

0.10


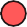

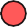

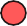

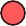

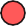

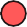

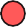

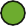

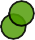

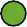

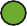

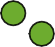


0.05

NMDS2

0.00

−0.05

−0.3 −0.2 −0.1 0.0 0.1 0.2

# NMDS1


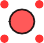
Forastero
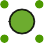
Hybrid

##PERMANOVA

**adonis**(dados2**~**varieties, permutations = 999)

##

## Call:

## adonis(formula = dados2 ~ varieties, permutations = 999) ##

0.00

0.04

## Permutation: free

## Number of permutations: 999 ##

## Terms added sequentially (first to last) ##

## Df SumsOfSqs MeanSqs F.Model R2 Pr(>F) ## varieties 1 0.005937 0.0059372 0.47666 0.0382 0.598

## Residuals 12 0.149473 0.0124561 0.9618

## Total 13 0.155410 1.0000

##Regarding all variables (transcriptomes), no difference ... PERMDISP <- **betadisper**(**vegdist**(dados2), varieties)

**plot**(PERMDISP)

**PERMDISP**

−0.15 −0.10 −0.05 0.00 0.05 0.10

Forastero

Hybrid

PCoA 2

−0.08

−0.04

PCoA 1

method = "bray"

**anova**(PERMDISP) ## Analysis of Variance Table

##

## Response: Distances

## Df Sum Sq Mean Sq F value Pr(>F) ## Groups 1 0.0002226 0.00022264 0.1169 0.7383

## Residuals 12 0.0228466 0.00190388
