# Supplementary material for: Unraveling potential enzymes and their functional role in fine cocoa beans fermentation using temporal shotgun metagenomics
Source: Front Microbiol. 2022 Nov 3;13:994524. doi: 10.3389/fmicb.2022.994524 (PMC9671152; doi:10.3389/fmicb.2022.994524)

**Supplementary Material 3**. NMDS plot of cocoa varieties samples in distinct fermentation times. Red points for Forastero (FOR) and Green points for MIX (mixture of two hybrid varieties.


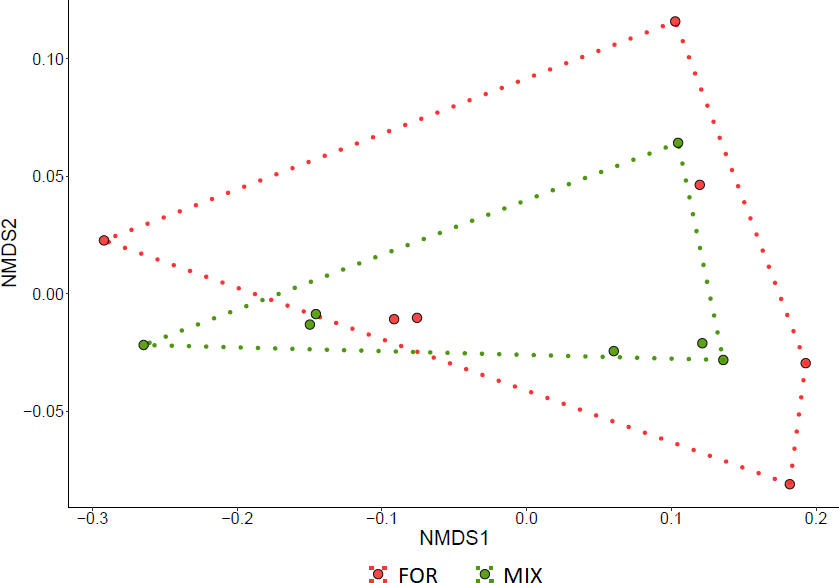

Supplement: Supplementary file 3 [file Table_3.DOCX]
