# Supplementary material for: Unraveling potential enzymes and their functional role in fine cocoa beans fermentation using temporal shotgun metagenomics
Source: Front Microbiol. 2022 Nov 3;13:994524. doi: 10.3389/fmicb.2022.994524 (PMC9671152; doi:10.3389/fmicb.2022.994524)
Supplement: Supplementary file 4 [file Table_4.DOCX]

**Supplementary Material 4.** Enzyme codes, yeast/bacteria genus, and the metabolism of lipids (LIP), carbohydrates (CHO), proteins (PTN), nucleic acids (NA), micromolecules and others (OTH) in fine cocoa fermentation.

| **Enzyme Code** | **Genus** | **Metabolism** |
| --- | --- | --- |
| **T1** |  |  |
| 1.1.1.37 | *Candida* | T1 - CHO |
| 1.13.11.20 | *Candida* | T1 - PTN |
| 1.14.14.17 | *Candida* | T1 - LIP |
| 1.2.1.31 | *Candida* | T1 - PTN |
| 1.2.1.95 | *Candida* | T1 - PTN |
| 1.3.3.6 | *Candida* | T1 - LIP |
| 2.6.1.13 | *Candida* | T1 - PTN |
| 2.7.1.1 | *Candida* | T1 - CHO |
| 2.7.1.105 | *Candida* | T1 - CHO |
| 2.7.1.11 | *Candida* | T1 - CHO |
| 2.7.11.1 | *Candida* | T1 - PTN |
| 2.7.11.24 | *Candida* | T1 - PTN |
| 2.7.4.2 | *Candida* | T1 - LIP |
| 3.1.3.16 | *Candida* | T1 - PTN |
| 3.1.3.46 | *Candida* | T1 - CHO |
| 3.2.1.14 | *Candida* | T1 - CHO |
| 3.2.1.18 | *Candida* | T1 - LIP |
| 3.2.1.39 | *Candida* | T1 - CHO |
| 4.2.1.22 | *Candida* | T1 - PTN |
| 5.1.3.15 | *Candida* | T1 - CHO |
| 6.1.1.22 | *Candida* | T1 - PTN |
| 7.1.2.1 | *Candida* | T1 - CHO |
| 5.4.2.11 | *Curtobacterium* | T1 - CHO |
| 1.1.1.34 | *Acetobacter* | T1 - LIP |
| 1.13.11.20 | *Acetobacter* | T1 - PTN |

| 3.1.3.16 | *Acetobacter* | T1 - PTN |
| --- | --- | --- |
| 4.1.1.2 | *Acetobacter* | T1 - CHO |
| 4.2.1.104 | *Acetobacter* | T1 - OTH |
| 7.1.2.1 | *Acetobacter* | T1 - CHO |
| 1.13.11.20 | *Asaia* | T1 - PTN |
| 2.7.7.5 | *Aspergillus* | T1 - OTH |
| 2.7.7.53 | *Aspergillus* | T1 - NA |
| 3.1.3.16 | *Aspergillus* | T1 - PTN |
| 2.7.11.1 | *Bacillus* | T1 - PTN |
| 2.7.4.21 | *Barnettozyma* | T1 - LIP |
| 2.7.4.24 | *Barnettozyma* | T1 - LIP |
| 3.1.2.14 | *Barnettozyma* | T1 - LIP |
| 3.1.3.16 | *Barnettozyma* | T1 - PTN |
| 3.1.3.16 | *Brevundimonas* | T1 - PTN |
| 2.4.2.1 | *Cronobacter* | T1 - NA |
| 3.1.3.16 | *Curtobacterium* | T1 - PTN |
| 1.1.1.37 | *Frateuria* | T1 - CHO |
| 1.13.11.20 | *Frateuria* | T1 - PTN |
| 1.14.14.35 | *Frateuria* | T1 - OTH |
| 1.2.1.70 | *Frateuria* | T1 - OTH |
| 2.7.1.21 | *Frateuria* | T1 - NA |
| 2.7.11.1 | *Frateuria* | T1 - PTN |
| 3.1.3.16 | *Frateuria* | T1 - PTN |
| 3.5.3.1 | *Frateuria* | T1 - PTN |
| 3.5.3.4 | *Frateuria* | T1 - NA |
| 5.4.2.11 | *Frateuria* | T1 - CHO |
| 6.1.1.22 | *Frateuria* | T1 - PTN |
| 1.13.11.20 | *Gluconobacter* | T1 - PTN |
| 2.7.1.21 | *Gluconobacter* | T1 - NA |
| 3.5.3.1 | *Gluconobacter* | T1 - PTN |

| 4.1.1.2 | *Gluconobacter* | T1 - CHO |
| --- | --- | --- |
| 4.2.1.104 | *Gluconobacter* | T1 - OTH |
| 7.1.2.1 | *Gluconobacter* | T1 - CHO |
| 1.1.1.34 | *Hanseniaspora* | T1 - LIP |
| 1.1.1.37 | *Hanseniaspora* | T1 - CHO |
| 1.1.1.8 | *Hanseniaspora* | T1 - LIP |
| 1.14.14.17 | *Hanseniaspora* | T1 - LIP |
| 1.2.1.31 | *Hanseniaspora* | T1 - PTN |
| 1.2.1.95 | *Hanseniaspora* | T1 - PTN |
| 1.4.1.14 | *Hanseniaspora* | T1 - PTN |
| 2.4.2.1 | *Hanseniaspora* | T1 - NA |
| 2.5.1.21 | *Hanseniaspora* | T1 - LIP |
| 2.6.1.13 | *Hanseniaspora* | T1 - PTN |
| 2.7.1.1 | *Hanseniaspora* | T1 - CHO |
| 2.7.1.105 | *Hanseniaspora* | T1 - CHO |
| 2.7.1.11 | *Hanseniaspora* | T1 - CHO |
| 2.7.1.20 | *Hanseniaspora* | T1 - NA |
| 2.7.1.36 | *Hanseniaspora* | T1 - LIP |
| 2.7.11.1 | *Hanseniaspora* | T1 - PTN |
| 2.7.11.24 | *Hanseniaspora* | T1 - PTN |
| 2.7.4.2 | *Hanseniaspora* | T1 - LIP |
| 2.7.4.21 | *Hanseniaspora* | T1 - LIP |
| 2.7.4.24 | *Hanseniaspora* | T1 - LIP |
| 2.7.6.2 | *Hanseniaspora* | T1 - OTH |
| 2.7.7.5 | *Hanseniaspora* | T1 - OTH |
| 2.7.7.53 | *Hanseniaspora* | T1 - NA |
| 2.7.8.11 | *Hanseniaspora* | T1 - LIP |
| 3.1.2.14 | *Hanseniaspora* | T1 - LIP |
| 3.1.3.16 | *Hanseniaspora* | T1 - PTN |
| 3.1.3.46 | *Hanseniaspora* | T1 - CHO |

| 3.2.1.14 | *Hanseniaspora* | T1 - CHO |
| --- | --- | --- |
| 3.2.1.18 | *Hanseniaspora* | T1 - LIP |
| 3.2.1.39 | *Hanseniaspora* | T1 - CHO |
| 3.5.3.1 | *Hanseniaspora* | T1 - PTN |
| 3.5.3.4 | *Hanseniaspora* | T1 - NA |
| 4.1.1.21 | *Hanseniaspora* | T1 - NA |
| 4.1.1.33 | *Hanseniaspora* | T1 - LIP |
| 4.2.1.22 | *Hanseniaspora* | T1 - PTN |
| 5.1.3.15 | *Hanseniaspora* | T1 - CHO |
| 5.4.2.11 | *Hanseniaspora* | T1 - CHO |
| 6.1.1.22 | *Hanseniaspora* | T1 - PTN |
| 7.1.2.1 | *Hanseniaspora* | T1 - CHO |
| 2.7.1.21 | *Klebsiella* | T1 - NA |
| 3.1.3.16 | *Klebsiella* | T1 - PTN |
| 3.2.1.18 | *Klebsiella* | T1 - LIP |
| 2.7.1.11 | *Kodamaea* | T1 - CHO |
| 1.13.11.20 | *Komagataeibacter* | T1 - PTN |
| 3.2.1.18 | *Komagataeibacter* | T1 - LIP |
| 4.1.1.2 | *Komagataeibacter* | T1 - CHO |
| 4.2.1.104 | *Komagataeibacter* | T1 - OTH |
| 1.1.1.34 | *Lactobacillus* | T1 - LIP |
| 2.7.1.21 | *Lactobacillus* | T1 - NA |
| 2.7.1.36 | *Lactobacillus* | T1 - LIP |
| 2.7.4.2 | *Lactobacillus* | T1 - LIP |
| 2.7.6.2 | *Lactobacillus* | T1 - OTH |
| 2.8.1.4 | *Lactobacillus* | T1 - OTH |
| 3.1.3.16 | *Lactobacillus* | T1 - PTN |
| 4.1.1.33 | *Lactobacillus* | T1 - LIP |
| 5.3.1.5 | *Lactobacillus* | T1 - CHO |
| 5.4.2.11 | *Lactobacillus* | T1 - CHO |

| 6.1.1.22 | *Lactobacillus* | T1 - PTN |
| --- | --- | --- |
| 6.3.1.20 | *Lactobacillus* | T1 - OTH |
| 3.1.3.16 | *Moraxella* | T1 - PTN |
| 2.7.6.2 | *Paenibacillus* | T1 - OTH |
| 3.2.1.14 | *Paenibacillus* | T1 - CHO |
| 1.1.1.34 | *Pantoea* | T1 - LIP |
| 1.1.1.37 | *Pantoea* | T1 - CHO |
| 1.13.11.20 | *Pantoea* | T1 - PTN |
| 1.14.14.35 | *Pantoea* | T1 - OTH |
| 1.2.1.70 | *Pantoea* | T1 - OTH |
| 2.4.2.1 | *Pantoea* | T1 - NA |
| 2.7.1.11 | *Pantoea* | T1 - CHO |
| 2.7.1.21 | *Pantoea* | T1 - NA |
| 2.7.11.1 | *Pantoea* | T1 - PTN |
| 2.8.1.4 | *Pantoea* | T1 - OTH |
| 3.1.3.16 | *Pantoea* | T1 - PTN |
| 3.2.1.14 | *Pantoea* | T1 - CHO |
| 3.2.1.18 | *Pantoea* | T1 - LIP |
| 4.1.1.2 | *Pantoea* | T1 - CHO |
| 4.7.1.1 | *Pantoea* | T1 - OTH |
| 5.1.3.15 | *Pantoea* | T1 - CHO |
| 5.3.1.5 | *Pantoea* | T1 - CHO |
| 5.4.2.11 | *Pantoea* | T1 - CHO |
| 6.1.1.22 | *Pantoea* | T1 - PTN |
| 6.3.1.20 | *Pantoea* | T1 - OTH |
| 3.5.3.4 | *Paraburkholderia* | T1 - NA |
| 1.1.1.34 | *Penicillium* | T1 - LIP |
| 2.7.1.1 | *Penicillium* | T1 - CHO |
| 2.7.1.105 | *Penicillium* | T1 - CHO |
| 2.7.11.1 | *Penicillium* | T1 - PTN |

| 3.1.3.16 | *Penicillium* | T1 - PTN |
| --- | --- | --- |
| 3.1.3.46 | *Penicillium* | T1 - CHO |
| 3.2.1.14 | *Penicillium* | T1 - CHO |
| 3.2.1.39 | *Penicillium* | T1 - CHO |
| 4.2.1.104 | *Penicillium* | T1 - OTH |
| 5.5.1.4 | *Penicillium* | T1 - CHO |
| 1.1.1.37 | *Pichia* | T1 - CHO |
| 2.7.1.1 | *Pichia* | T1 - CHO |
| 2.7.11.1 | *Pichia* | T1 - PTN |
| 2.7.7.5 | *Pichia* | T1 - OTH |
| 2.7.7.53 | *Pichia* | T1 - NA |
| 1.13.11.20 | *Pseudomonas* | T1 - PTN |
| 1.2.1.70 | *Pseudomonas* | T1 - OTH |
| 2.7.11.1 | *Pseudomonas* | T1 - PTN |
| 2.8.1.4 | *Pseudomonas* | T1 - OTH |
| 3.1.3.16 | *Pseudomonas* | T1 - PTN |
| 2.7.11.1 | *Rahnella* | T1 - PTN |
| 1.1.1.34 | *Rhizopus* | T1 - LIP |
| 1.14.14.17 | *Rhizopus* | T1 - LIP |
| 1.2.1.31 | *Rhizopus* | T1 - PTN |
| 1.2.1.95 | *Rhizopus* | T1 - PTN |
| 1.3.3.6 | *Rhizopus* | T1 - LIP |
| 2.4.2.1 | *Rhizopus* | T1 - NA |
| 2.7.1.1 | *Rhizopus* | T1 - CHO |
| 2.7.1.11 | *Rhizopus* | T1 - CHO |
| 2.7.11.1 | *Rhizopus* | T1 - PTN |
| 3.1.2.14 | *Rhizopus* | T1 - LIP |
| 3.1.3.16 | *Rhizopus* | T1 - PTN |
| 3.2.1.14 | *Rhizopus* | T1 - CHO |
| 3.2.1.39 | *Rhizopus* | T1 - CHO |

| 4.1.1.33 | *Rhizopus* | T1 - LIP |
| --- | --- | --- |
| 4.2.1.22 | *Rhizopus* | T1 - PTN |
| 5.5.1.4 | *Rhizopus* | T1 - CHO |
| 7.1.2.1 | *Rhizopus* | T1 - CHO |
| 2.7.11.1 | *Rhodanobacter* | T1 - PTN |
| 5.4.2.11 | *Rhodanobacter* | T1 - CHO |
| 3.2.1.14 | *Rouxiella* | T1 - CHO |
| 3.2.1.14 | *Salmonella* | T1 - CHO |
| 6.1.1.22 | *Saturnispora* | T1 - PTN |
| 3.1.3.16 | *Sphingobium* | T1 - PTN |
| 1.1.1.8 | *Wickerhamomyces* | T1 - LIP |
| 2.7.1.1 | *Wickerhamomyces* | T1 - CHO |
| 4.1.1.33 | *Wickerhamomyces* | T1 - LIP |
| **T2** |  |  |
| 1.13.11.53 | *Acetobacter* | T2 - PTN |
| 1.4.9.1 | *Acetobacter* | T2 - OTH |
| 2.3.1.51 | *Acetobacter* | T2 - LIP |
| 2.4.1.288 | *Acetobacter* | T2 - CHO |
| 3.5.4.5 | *Acetobacter* | T2 - NA |
| 4.2.1.129 | *Acetobacter* | T2 - LIP |
| 5.4.99.17 | *Acetobacter* | T2 - LIP |
| 3.5.4.5 | *Bacteroides* | T2 - NA |
| 3.6.1.22 | *Bacteroides* | T2 - OTH |
| 1.2.1.71 | *Brevundimonas* | T2 - PTN |
| 3.1.3.3 | *Brevundimonas* | T2 - PTN |
| 3.6.1.22 | *Brevundimonas* | T2 - OTH |
| 7.3.2.3 | *Brevundimonas* | T2 - OTH |
| 1.13.11.53 | *Frateuria* | T2 - PTN |
| 2.4.1.80 | *Frateuria* | T2 - LIP |
| 3.5.4.5 | *Frateuria* | T2 - NA |
| 3.6.1.22 | *Frateuria* | T2 - OTH |
| 4.2.1.129 | *Frateuria* | T2 - LIP |
| 5.4.99.17 | *Frateuria* | T2 - LIP |
| 3.6.1.22 | *Fulvimonas* | T2 - OTH |
| 1.13.11.53 | *Gluconobacter* | T2 - PTN |
| 2.4.1.80 | *Gluconobacter* | T2 - LIP |
| 3.1.3.3 | *Gluconobacter* | T2 - PTN |

| 3.5.4.5 | *Gluconobacter* | T2 - NA |
| --- | --- | --- |
| 4.2.1.129 | *Gluconobacter* | T2 - LIP |
| 5.4.99.17 | *Gluconobacter* | T2 - LIP |
| 2.3.1.51 | *Hanseniaspora* | T2 - LIP |
| 2.3.3.10 | *Hanseniaspora* | T2 - LIP |
| 2.4.1.80 | *Komagataeibacter* | T2 - LIP |
| 3.1.3.3 | *Komagataeibacter* | T2 - PTN |
| 3.5.4.5 | *Komagataeibacter* | T2 - NA |
| 4.2.1.129 | *Komagataeibacter* | T2 - LIP |
| 5.4.99.17 | *Komagataeibacter* | T2 - LIP |
| 2.4.1.80 | *Kozakia* | T2 - LIP |
| 3.1.3.3 | *Kozakia* | T2 - PTN |
| 3.6.1.22 | *Moraxella* | T2 - OTH |
| 1.2.1.71 | *Pantoea* | T2 - PTN |
| 2.3.1.51 | *Pantoea* | T2 - LIP |
| 3.1.3.3 | *Pantoea* | T2 - PTN |
| 3.5.4.5 | *Pantoea* | T2 - NA |
| 3.6.1.22 | *Pantoea* | T2 - OTH |
| 7.3.2.3 | *Pantoea* | T2 - OTH |
| 1.2.1.71 | *Pseudomonas* | T2 - PTN |
| 3.1.3.3 | *Pseudomonas* | T2 - PTN |
| 3.6.1.22 | *Pseudomonas* | T2 - OTH |
| 7.3.2.3 | *Pseudomonas* | T2 - OTH |
| 3.5.4.5 | *Sphingobium* | T2 - NA |
| 2.4.1.80 | *Sphingomonas* | T2 - LIP |
| 3.5.4.5 | *Sphingomonas* | T2 - NA |
| 3.6.1.22 | *Sphingomonas* | T2 - OTH |
| 2.4.1.80 | *Tanticharoenia* | T2 - LIP |
| **T3** |  |  |
| 2.4.1.21 | *Acetobacter* | T3 - CHO |
| 3.2.1.141 | *Acetobacter* | T3 - CHO |
| 3.5.1.1 | *Acetobacter* | T3 - PTN |
| 5.4.99.15 | *Acetobacter* | T3 - CHO |
| 2.4.1.21 | *Alistipes* | T3 - CHO |
| 2.4.1.21 | *Alistipes* | T3 - CHO |
| 2.4.1.21 | *Gluconobacter* | T3 - CHO |
| 3.2.1.141 | *Gluconobacter* | T3 - CHO |
| 3.5.1.1 | *Hanseniaspora* | T3 - PTN |
| 2.4.1.21 | *Komagataeibacter* | T3 - CHO |
| 3.2.1.141 | *Komagataeibacter* | T3 - CHO |
| 3.5.1.1 | *Komagataeibacter* | T3 - PTN |

| 5.4.99.15 | *Komagataeibacter* | T3 - CHO |
| --- | --- | --- |
| 2.4.1.21 | *Kozakia* | T3 - CHO |
| 2.4.1.21 | *Pantoea* | T3 - CHO |
| 3.5.1.1 | *Pantoea* | T3 - PTN |
| 2.4.1.21 | *Pseudomonas* | T3 - CHO |
| 3.2.1.141 | *Pseudomonas* | T3 - CHO |
| 3.5.1.1 | *Pseudomonas* | T3 - PTN |
| 5.4.99.15 | *Pseudomonas* | T3 - CHO |
| 2.4.1.21 | *Sphingomonas* | T3 - CHO |
